# Identification, Recovery, and Refinement of Hitherto Undescribed Population-Level Genomes from the Human Gastrointestinal Tract

**DOI:** 10.3389/fmicb.2016.00884

**Published:** 2016-06-21

**Authors:** Cedric C. Laczny, Emilie E. L. Muller, Anna Heintz-Buschart, Malte Herold, Laura A. Lebrun, Angela Hogan, Patrick May, Carine de Beaufort, Paul Wilmes

**Affiliations:** ^1^Luxembourg Centre for Systems Biomedicine, University of LuxembourgBelvaux, Luxembourg; ^2^Integrated Biobank of LuxembourgLuxembourg, Luxembourg; ^3^Centre Hospitalier de LuxembourgLuxembourg, Luxembourg

**Keywords:** metagenome, binning, genome recovery, refinement, reference genomes

## Abstract

Linking taxonomic identity and functional potential at the population-level is important for the study of mixed microbial communities and is greatly facilitated by the availability of microbial reference genomes. While the culture-independent recovery of population-level genomes from environmental samples using the binning of metagenomic data has expanded available reference genome catalogs, several microbial lineages remain underrepresented. Here, we present two reference-independent approaches for the identification, recovery, and refinement of hitherto undescribed population-level genomes. The first approach is aimed at genome recovery of varied taxa and involves multi-sample automated binning using CANOPY CLUSTERING complemented by visualization and human-augmented binning using VIZBIN *post hoc*. The second approach is particularly well-suited for the study of specific taxa and employs VIZBIN *de novo*. Using these approaches, we reconstructed a total of six population-level genomes of distinct and divergent representatives of the Alphaproteobacteria class, the Mollicutes class, the Clostridiales order, and the Melainabacteria class from human gastrointestinal tract-derived metagenomic data. Our results demonstrate that, while automated binning approaches provide great potential for large-scale studies of mixed microbial communities, these approaches should be complemented with informative visualizations because expert-driven inspection and refinements are critical for the recovery of high-quality population-level genomes.

## Introduction

Substantial efforts have recently been undertaken for the in-depth structural and functional characterization of human-derived microbiota from various body sites (Qin et al., 2010; Huttenhower et al., 2012; Methé et al., 2012). These efforts largely involve the use of microbial isolate genome sequences (Lagier et al., 2012; Rajilić-Stojanović and de Vos, 2014) or population-level genomes recovered following the “binning” of metagenomic data (Di Rienzi et al., 2013; Sharon et al., 2013; Alneberg et al., 2014; Nielsen et al., 2014). Importantly, the term “population-level genome” indicates that the genomic complements recovered from metagenomic data will typically be derived from a mixture (population) of closely related microorganisms in which individuals are not expected to be clonal (Kunin et al., 2008), thus resulting in composite genomic reconstructions. Obtaining population-level genome resolution, e.g., at the genus- or species-levels, allows linkage of taxonomic identity and function at the level of individual populations (Dick et al., 2009; Albertsen et al., 2013; Muller et al., 2014).

Unsupervised binning approaches, as opposed to their supervised counterparts, are independent of prior information and exploit data-inherent characteristics, e.g., genomic signatures based on oligonucleotide frequencies and/or sequence abundance information (Dick et al., 2009; Albertsen et al., 2013; Alneberg et al., 2014; Laczny et al., 2014; Nielsen et al., 2014; Wu et al., 2014; Kang et al., 2015). These reference-independent binning approaches may further be subdivided into automated approaches (Nielsen et al., 2014; Wu et al., 2014), e.g., CANOPY CLUSTERING, and user-driven approaches (Dick et al., 2009; Laczny et al., 2014, 2015), e.g., VIZBIN. For the former, minimal, if any, input by a human user is needed, whereas for the latter a low-dimensional representation, typically in two dimensions, allows for human input in the cluster definition process. The automated CANOPY CLUSTERING employs sequence abundance covariation across a set of multiple samples and has been used for the large-scale recovery of population-level genomes from the human gastrointestinal tract (GIT; Nielsen et al., 2014). In the user-driven VIZBIN, bins are represented as two-dimensional sequence cluster structures and resolved using human pattern recognition capabilities (Laczny et al., 2014, 2015). While the automation of the clustering process typically leads to an increased throughput in the recovery of population-level genomes from metagenomic data, suboptimal clusters might be created and should be manually refined (Laczny et al., 2015).

Despite previous efforts to further expand reference genome catalogs, individual microbial lineages might have been missed even in deeply studied environments such as the human GIT. Unsupervised binning approaches are particularly pertinent for the recovery of genomes derived from members of hitherto undescribed microbial lineages due to their independence of prior information, especially if no closely related representatives have yet been recovered. An example of such a novel lineage discovered following the binning of metagenomic data are the Melainabacteria which occur in environmental as well as human-derived samples (Di Rienzi et al., 2013; Soo et al., 2014). While this lineage was reported in earlier 16S rRNA gene-based studies of the human GIT (Ley et al., 2005), sets of complete or partial population-level genomes were only recently recovered from metagenomic data derived from human and koala fecal samples (Di Rienzi et al., 2013; Soo et al., 2014). The Melainabacteria-lineage was originally proposed as a sister-phylum to the Cyanobacteria (Di Rienzi et al., 2013) but Soo et al. (2014) subsequently suggested that it represents a non-photosynthetic class within the Cyanobacteria phylum instead.

Here, we present two approaches for the identification, recovery, and refinement of hitherto undescribed population-level genomes without the need for *a priori* knowledge in the form of reference genomes. These approaches involving automated binning using CANOPY CLUSTERING and/or visualization and human-augmented binning using VIZBIN were applied to human GIT-derived metagenomic data from a multiplex family study of type 1 diabetes mellitus (MuSt). VIZBIN was first applied *post hoc* (automatically generated clusters were inspected and manually refined using VIZBIN) and using this approach one automatically identified cluster was found to be a mixture of at least three distinct organisms. Second, given the recent discovery of the Melainabacteria class, VIZBIN was used to explore whether genomic complements of further, hitherto undescribed representatives could be recovered by the *de novo* application of VIZBIN, i.e., without prior automated clustering. Overall, a total of six almost complete or partial population-level genomes from the Alphaproteobacteria class, the Mollicutes class, the Clostridiales order, and the Melainabacteria class were recovered thereby extending existing reference genome catalogs. The reconstructed, high-quality population-level genomes will be valuable for the successful interpretation of additional multi-omic data from the human GIT. Moreover, we would expect our approach to be applicable for the reconstruction of high-quality population-level genomes from metagenomic data derived from other, less well-characterized environments.

## Materials and Methods

### Sample Collection, Processing, and Metagenomic Sequencing

#### Study Context

The multiplex family study of type 1 diabetes mellitus is a Luxembourg-based, observational study of selected family groups of two or three generations in which there are multiple incidents of type 1 diabetes mellitus (T1DM). Fecal samples were collected at different time points from patients with T1DM and healthy family members. A total of 55 fecal samples were collected from 10 patients with T1DM and 10 healthy family members. The generated metagenomic data is used herein for the identification and recovery of hitherto undescribed microbial population-level genomes, independent of disease burden. The study was approved by the Comité d’Ethique de Recherche (CNER; Reference: 201110/05) and the National Commission for Data Protection in Luxembourg. Written informed consent was obtained from all subjects enrolled in the study.

#### Stool Sampling

Fecal samples were self-collected and immediately frozen on dry ice at three time points (if bowel movement permitted on day of scheduled sampling) at intervals between 4 and 8 weeks.

#### Extraction of DNA from Fecal Samples

DNA was extracted from frozen subsamples of 150 mg after pre-treatment of the weighed subsamples with 1.5 ml RNAlater ICE (Life Technologies) at -20°C over night. The faeces-RNAlater ICE mixtures were homogenized by bead-beating (Roume et al., 2013). Differential centrifugation and extraction using All-In-One kit (Norgen Biotek) were performed according to Roume et al. (2013). DNA fractions were further supplemented with DNA extracted from 200 mg subsamples using the MOBIO Power Soil Kit according to the manufacturer’s instructions.

#### Library Preparation and Sequencing

Libraries with an insert size of 350 base pair (bp) were constructed from metagenomic DNA following fragmentation by sonification (Covaris), end-repair, adenylation, adapter ligation, and amplification of adapter-ligated DNA fragments using appropriate enzymes (Enzymatics). Library amplification and cluster generation were performed using TruSeq PE Cluster Kit V3–cBot–HS (Illumina). The resulting flow cells were sequenced on a HiSeq2000 system (Illumina) generating 101 bp paired-end reads. Sequencing was performed by BGI (Hong Kong, China).

### Preprocessing of the Metagenomic Data

The per-sample metagenomic paired-end sequencing data in FASTQ format was processed using the MOCAT trimming and quality filtering step (MOCAT.pl -rtf) and the parameters used were as follows: readtrimfilter_length_cutoff = 40 readtrim filter_qual_cutoff = 20 readtrimfilter_use_sanger_scale = yes readtrimfilter_trim_5_prime = yes readtrimfilter_use_precalc_5prime_trimming = no (Kultima et al., 2012). The preprocessed reads were then mapped onto the human reference genome (hg19) using the MOCAT screening step (MOCAT.pl -s hg19) and using the following parameters: screen_length_cutoff = 30 screen_percent_cutoff = 90 screen_soap_seed_length = 30 screen_soap_max_mm = 10 screen_soap_cmd = -M 4 screen_save_format = sam and SOAPALIGNER v2.21 (Li R. et al., 2009). The preprocessing resulted in two sets of reads in FASTQ format (human and non-human) consisting each of paired-end and single-end reads. The human reads were discarded. A schematic overview of the individual steps is provided in **Supplementary Figure S1**.

### Assembly of the Metagenomic Data

The preprocessed, non-human, paired-end reads of each sample were assembled separately using IDBA-UD (Peng et al., 2012). More specifically, the reads were converted from FASTQ to FASTA format using the FQ2FA script (fq2fa --merge --filter) provided by IDBA-UD. Subsequently, IDBA-UD was applied using its pre-error-correction step for read error correction (idba_ud --pre_correction). The resulting contigs were extended using the paired-end and single-end reads not used by IDBA-UD using the VELVET assembly tool (Zerbino and Birney, 2008). First, paired-end reads were mapped onto the previously assembled contigs using SOAP (-r 2 -M 4 -l 30 -v 10 -p 8 -u unmapped.fa) and “unmapped” reads were identified. Then, the unused single-end reads (IDBA-UD only supports paired-end reads) were combined with the unmapped reads, and cd-hit-dup from the CD-HIT software suite was used to remove duplicate reads (Fu et al., 2012). The IDBA-UD-based contigs were provided as long-read input to VELVET v1.2.07 with the following parameters for velveth: -long contig.fa, for velvetg: -conserveLong yes, and over a range of *k*-mer sizes (27, 31, 35, 39, 43, 47, 51, 55, 59, 63). The resulting contigs from the assemblies using different *k*-mer sizes and the IDBA-UD-based initial set of contigs were pooled and clustered using cd-hit-est (parameter: -c 0.99) to remove redundancy. MINIMUS2 (AMOS genome assembly software suite v3.1) was used to join and extend the clustered contigs based on the detection of sequence overlaps (Treangen et al., 2011). Gene prediction was performed on the final set of contigs using PRODIGAL v2.60 (parameter: -p meta; Hyatt et al., 2012). A schematic overview of the individual steps is provided in **Supplementary Figure S1**.

### Automated Clustering Using CANOPY CLUSTERING

Genes from the individual assemblies were pooled and genes with a sequence length <100 nt were discarded. The remaining genes were made non-redundant by applying cd-hit-est (Li and Godzik, 2006; Fu et al., 2012) to collapse sequences with 95% sequence identity over 90% of the shorter sequence (-c 0.95 -aS 0.9). BOWTIE2 v2.0.2 was used to map the preprocessed reads for the individual samples to the gene catalog. The resulting SAM files were sorted using SAMTOOLS v0.1.19 and converted to BAM format. BEDTOOLS v2.18.1 genomeCoverageBed and AWK were used to compute the per-sample fold-coverage for each catalog-gene. Genes with a fold-coverage <2× in all of the 55 samples were discarded to reduce the data amount and to limit the runtime on the University of Luxembourg’s High Performance Computing platform. Put differently, a gene was preserved if its fold-coverage was ≥2× in at least one of the 55 samples. CANOPY CLUSTERING was run with the following parameters: --max_canopy_dist 0.1 --max_close_dist 0.4 --max_merge_dist 0.1 --min_step_dist 0.005 --stop_fraction 1 and 30 threads (-n 30). Following the original definition, the resulting co-abundance gene groups (CAGs) are referred to as metagenomic species (MGS) if they contained at least 700 genes (Nielsen et al., 2014).

### Completeness and Contamination Estimation of Population-Level Genomes

A set of 107 genes found in single copy in 95% of sequenced bacterial genomes (essential genes) was used to assess the degrees of completeness and contamination of individual population-level genomes (Dupont et al., 2012). Peptide sequences of the *in silico* predicted genes were screened against the hidden Markov models (HMMs) of the essential genes^1^ (Albertsen et al., 2013) using HMMER v3.1b1 hmmsearch with parameters: --cut_tc –notextw (Eddy, 2007). High quality population-level genomes are characterized by high levels of completeness (high fraction of essential genes recovered) and low levels of contamination (low number of duplicated essential genes).

### Taxonomic Characterization

Complementary approaches for the taxonomic characterization of the bins were used. These included two approaches based on protein-coding genes [PHYLOPHLAN (Segata et al., 2013) and AMPHORA2 (Wu and Scott, 2012)] and a whole genome-based approach [BLAST (Altschul et al., 1990, 1997; Zhang et al., 2000; Wheeler et al., 2007) + MEGAN (Huson et al., 2007)]. For individual genomic complements, PHYLOPHLAN provides a consensus taxonomic classification, whereas AMPHORA2 returns taxonomic classifications for each gene separately out of a set of phylogenetic marker genes. While the whole genome-based approach queries a continuously updated database at the NCBI and may thus potentially be more specific, the two protein-coding gene-based approaches are more robust with respect to the taxonomic characterization of organisms without closely related representatives in a database.

#### PHYLOPHLAN

The translated peptide sequences for each gene (see sections “Assembly of the Metagenomic Data” and “Automated Clustering Using CANOPY CLUSTERING”) were prepared for PHYLOPHLAN by ensuring unique peptide sequence identifiers and removing the asterisk symbol (if present) at the ends of the sequences. PHYLOPHLAN’s impute option (-t) was used for taxonomic assignment of individual populations and 12 threads were used (--nproc 12).

#### AMPHORA2

The default parameters of AMPHORA2 were used.

#### BLAST + MEGAN

Online BLAST searches were carried out on the “Nucleotide collection (nr/nt)” database. “Uncultured/environmental sample sequences” were excluded and “bacteria (taxid:2)” was specified as “Organism.” The “Max target sequences” were set to 10. All other parameters were left at their default values. The results were downloaded and imported into MEGAN using the lowest common ancestor (LCA)-option to obtain per-sequence taxonomic classifications.

### Whole Genome-Based Comparisons

The online average nucleotide identity (ANI) calculator^2^ was used for determining the ANI values between genomic complements (Goris et al., 2007). Genome pairs with ANI values >95% were considered to belong to the same species (Goris et al., 2007).

### Cyanobacteria-Like Sequence Groups

#### Identification of Marker Genes

AMPHORA2 (Wu and Scott, 2012) was used to identify and to taxonomically classify phylogenetic marker genes among the genes encoded by the *de novo* assembled contigs of the 55 MuSt metagenomic datasets (see section “Assembly of the Metagenomic Data”). All genes annotated to belong to the Cyanobacteria phylum with an associated confidence value ≥75% were retained. The DNA-directed RNA polymerase subunit beta (*rpoB*) genes were selected for further analyses, as they represented the largest set of marker genes annotated as cyanobacterial. Incomplete *rpoB* gene predictions, i.e., those lacking a start or a stop codon according to the gene prediction, were discarded. The remaining *rpoB* genes were then clustered according to sequence similarity using cd-hit-est at 95% sequence identity over 50% of the shorter sequences (-c 0.95 -aS 0.5; Li and Godzik, 2006; Fu et al., 2012). The resulting sequence clusters are referred to as “Cyanobacteria-like sequence groups (CLSGs)” and the respective representative genes as “CLSG marker genes.”

#### Coverage Computation

The preprocessed reads of each sample were individually mapped to the CLSG genes using BOWTIE2 v2.0.2. The resulting SAM files were sorted using SAMTOOLS v0.1.19 and converted to BAM format (Li H. et al., 2009). Per-CLSG gene fold-coverages for each sample were computed using BEDTOOLS v2.23.0 genomeCoverageBed (Quinlan and Hall, 2010) and AWK.

### Construction of Phylogenetic Trees Using *rpoB*

The *rpoB* gene was used for the construction of phylogenetic trees as it represents an alternative to the ribosomal rRNA genes for phylogenetic analyses (Case et al., 2007; Bondoso et al., 2013). For the MGS (see section “Automated Clustering Using CANOPY CLUSTERING”), the NCBI’s MOLE-BLAST webservice^3^ was used which includes a database search to retrieve the most closely related sequences, thus accounting for the varied MGS taxa, i.e., of diverse phylogenetic origin. In contrast, for the CLSG genomes (see section “Cyanobacteria-Like Sequence Groups”), melainabacterial *rpoB* genes were manually extracted from published melainabacterial genomes.

#### MGS

The NCBI’s MOLE-BLAST webservice^3^ was used to query the *rpoB* gene sequences encoded by the VIZBIN-refined MGS population-level genomes against the “Nucleotide collection (nr/nt)” database. “Uncultured/environmental sample sequences” were excluded and “Bacteria” was specified as an “Entrez Query.” The “Number of database sequences” was set to 10. In brief, MOLE-BLAST generally works as follows. In the first step, the query sequences are grouped by locus using BLAST (Altschul et al., 1997). Second, a BLAST database search is performed to identify each query’s nearest-neighbor target sequences. A multiple sequence alignment is subsequently computed for each locus, including the query sequences and their nearest neighbors, using MUSCLE (Edgar, 2004). MOLE-BLAST then computes a phylogenetic tree for each locus multiple sequence alignment using Neighbor Joining (Saitou and Nei, 1987) or Fast Minimum Evolution (Desper and Gascuel, 2004).

#### CLSGs and Melainabacteria

Gut-derived and environment-derived Melainabacteria genomes from Di Rienzi et al. (2013) were downloaded^4^ Gut-derived Melainabacteria genomes from Soo et al. (2014) were downloaded from JGI IMG/ER under the accession numbers 2523533517 (Zag_1), 2531839741 (Zag_111), 2523533519 (Zag_221), 2522572068 (MH_37). The *rpoB* genes were identified using PROKKA (Seemann, 2014) and their nucleotide sequences were extracted. All *rpoB* gene sequences in the publicly available Melainabacteria genomes and the CLSG genes identified in this work were submitted to phylogeny.fr using the “One Click” mode^5^ (Dereeper et al., 2008). The option “Use the Gblocks program to eliminate poorly aligned positions and divergent regions” was enabled. The alignment of the *rpoB* gene sequences was computed using MUSCLE (Edgar, 2004) and automatically curated using GBLOCKS (min. seq. for flank pos.: 85%; max. contig. Non-conserved pos.: 8; min. block length: 10; gaps in final blocks: no; Castresana, 2000). PHYML including the approximate Likelihood-Ratio Test (aLRT) was used to compute the phylogeny (model: default; statistical test: alrt; number of categories: 4, gamma: estimated; invariable sites: estimated; remove gaps: enabled; Guindon and Gascuel, 2003; Anisimova and Gascuel, 2006; Guindon et al., 2010). The tree was saved in Newick-format and rendered using EVOLVIEW (Zhang et al., 2012). Coloring of the resulting tree was performed manually in ADOBE ILLUSTRATOR.

### Visualization and Binning Using VIZBIN

Contigs from the respective samples were used as input for VIZBIN (Laczny et al., 2015). The resulting two-dimensional embeddings were employed to select bins of interest. If not stated otherwise, reconstructed metagenomic sequence fragments <1,000 nt were omitted from the visualization and binning using VIZBIN (Laczny et al., 2015). Remaining sequences longer than 5,000 nt were iteratively cut into segments (chunks) of 5,000 nt as long as the remaining sequence was at least 5,000 nt long. Otherwise the entire (remaining) sequence was used. The creation of sequence chunks helps to mitigate effects of variations in assembly quality on the visualization: well-recovered genomes are assembled in longer and fewer contigs than other genomes. However, long-assembled genomes would, without the creation of chunks, be represented by only a small number of points. This step can thus be considered as a means to normalize sequence cluster size and density for improved cluster identification and delineation. The resulting sequences were used as input for VIZBIN. Additional information was added to the visualizations on a per-case basis. Generally, if coverage information was used, the opaqueness value (alpha) of each point was determined based on the natural logarithm of the corresponding fold-coverage value and provided as the “coverage” annotation option in VIZBIN. Particular sequences of interest were highlighted using either the “label” annotation type (distinct color and shape per individual label) in the case of the manual refinement of automatically generated MGS or the “isMarker” annotation type (star-shape; “beacon contig”) in the case of the manually defined CLSG bins.

### Re-assembly and Analysis of Recovered Population-Level Genomes

The preprocessed reads (pairs and singletons) from the sample with the highest average contig fold-coverage for each population-level genome were aligned to the contigs of the population-level genome using BOWTIE2 v.2.2.2 with default parameters. Contigs with exceptionally high or low fold-coverage, i.e., outliers, were identified and excluded. More specifically, a contig was considered an outlier if the absolute value of the modified *Z*-score was greater than 3.5 (Iglewicz and Hoaglin, 1993). The reads (pairs and singletons) from the preserved contigs were recruited using SAMTOFASTQ from PICARD v.1.130^6^ and assembled using SPADES v.3.1.0 (Bankevich et al., 2012) using the “careful” option.

#### RAST-Based Annotation and Accession

Functional annotation of the re-assembled genomes was performed using the RAST webservice^7^ (Aziz et al., 2008; Overbeek et al., 2014). The respective annotation results, including the original genomes, are accessible under the following accession IDs via a guest account: MGS00153 – 6666666.163363, MGS00248 – 6666666.163364, MGS00113-CG02 – 6666666.163361, CLSG01 – 6666666.163354, CLSG02 – 6666666.163355, CLSG03 – 6666666.155161. The genome analyses were performed by using automatically computed ‘Scenarios’ as well as by user-driven search of specific genes in the ‘Genome Browser’ of the RAST webservice. Gaps in nearly complete pathways or complexes were filled manually using a BLAST search of the missing genes.

#### Data Accession

The raw non-human metagenomic reads are deposited at the NCBI under the BioProject accession number PRJNA289586.

## Results and Discussion

**FIGURE 1:**
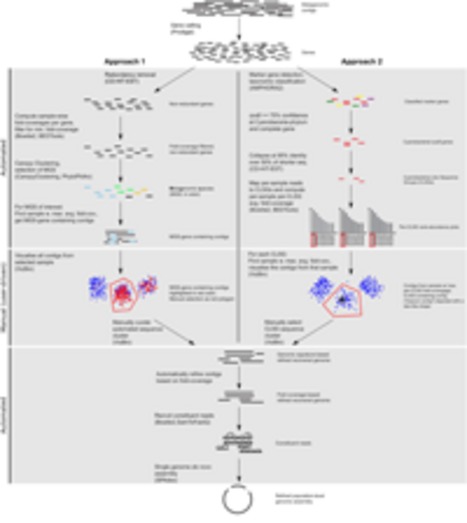
**Workflow scheme for the identification, recovery, and refinement of hitherto undescribed population-level genomes from metagenomic data.** Approach 1: the CANOPY CLUSTERING-based clusters are manually inspected and refined using VIZBIN. Approach 2: CLSGs are identified and used as beacon contigs to highlight the respective clusters. The main tools used at each step (see “Materials and Methods”) are listed in parentheses.

Here, we present the results of the application of our two approaches, involving CANOPY CLUSTERING and/or VIZBIN, for the identification, recovery, and refinement of population-level genomes from human GIT-derived metagenomic data of the MuSt project (**Figure 1**). The metagenomic data was preprocessed and assembled using a custom pipeline (**Supplementary Figure S1**). On average, 20,862,561 paired-end reads ± 608,594 (mean ± SD) remained after preprocessing per sample thus resulting in highly similar read library sizes across all samples. On average, 877,215 contigs were assembled (**Supplementary Table S1**).

### Inspection and Refinement of Automatically Generated Bins

CANOPY CLUSTERING requires the construction of a non-redundant gene catalog and exploits the cross-sample correlation of fold-coverages of genes in the catalog for bin (cluster) definition. Here, CANOPY CLUSTERING was first applied to the MuSt metagenomic dataset consisting of 55 samples from 20 individuals in total. A subset of the resulting clusters was subsequently inspected and refined using VIZBIN (**Figure 1**). This allowed an assessment whether CANOPY CLUSTERING could be used for initial cluster definition/identification and whether it would benefit from a *post hoc* application of VIZBIN for the recovery of hitherto undescribed population-level genomes from human GIT-derived metagenomic data.

The non-redundant gene catalog comprised 8,576,852 non-redundant genes. After filtering for low-fold-coverage genes (<fold-coverage < 2x per sample in all samples), the per-sample fold-coverage values of the remaining genes were aggregated into a 4,343,293-by-55 matrix serving as the input for CANOPY CLUSTERING. In total, 365 clusters containing at least 700 genes were returned by CANOPY CLUSTERING. Clusters with a minimum of 700 genes are referred to as “MGS” (Nielsen et al., 2014) and the following results focus on the 365 identified MGS. The degrees of completeness and contamination were assessed using the set of 107 essential genes (see “Materials and Methods”). The distribution of the numbers of different essential genes per MGS (reflecting completeness) exhibited two major modes, one around a total of 10 and one around a total of 100 essential genes per MGS (**Supplementary Figure S2**; top marginal distribution). The median was 47 essential genes (mean was 49.55), with 75% of the MGS having ≤85 of the 107 essential genes in at least one copy. The 365 MGS generally demonstrated a low degree of contamination (**Supplementary Figure S2**; right marginal distribution). More specifically, the median was three essential genes in multiple copies (mean was 9.1) and 75% of the MGS had ≤10 essential genes in multiple copies. However, an increase in the degree of contamination, i.e., an increase in the numbers of essential genes in multiple copies per MGS, could be observed with an increase in completeness (**Supplementary Figure S2**). This may be due to various reasons including suboptimal clustering or covarying microorganisms. In any case, suboptimal completeness and contamination results suggested that it could be promising to use a complementary approach, here VIZBIN, for the inspection and potential refinement of CANOPY CLUSTERING-based clusters.

**FIGURE 2:**
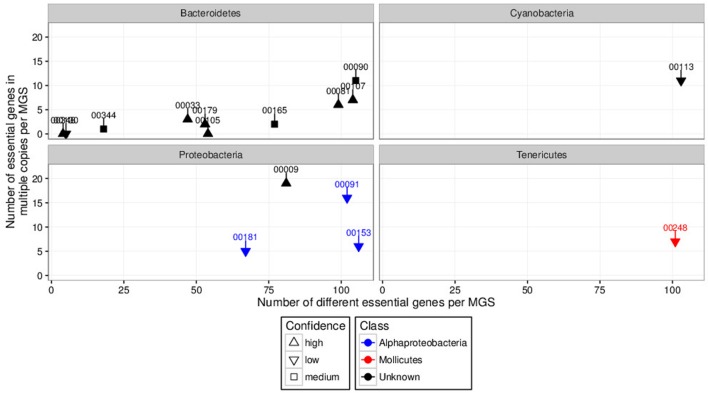
**Degrees of completeness (number of different essential genes per MGS; *x*-axis) and contamination (number of essential genes in multiple copies per MGS; *y*-axis) analyses results of automatically generated MGS with unknown order-level assignment grouped by phyla of interest.** Some MGS were unclassified at the class-level (black). Numbers above and connected to points indicate respective MGS identifiers. MGS00153, MGS00248, and MGS00113 were selected for inspection and manual refinement using VIZBIN. The “confidence levels” (high, medium, or low) of the individual taxonomic assignments were assigned by PHYLOPHLAN.

**FIGURE 3:**
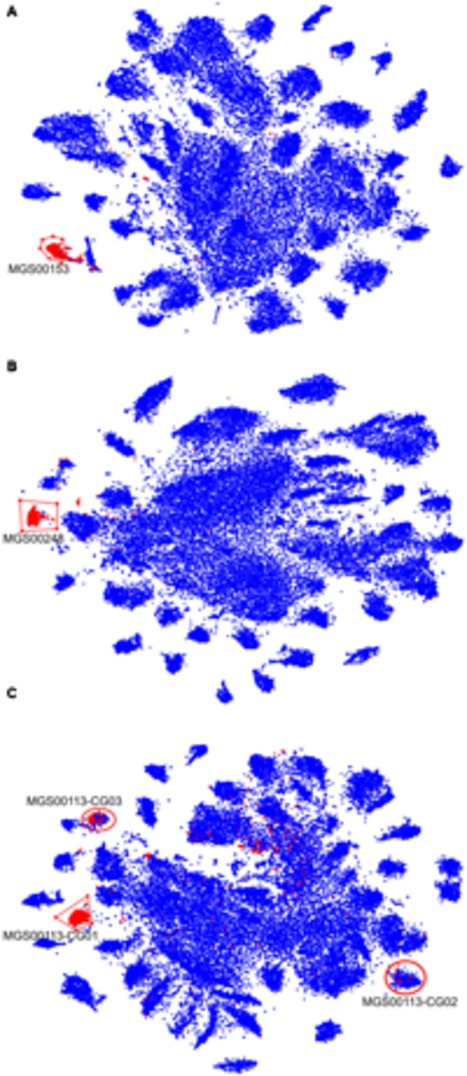
**VIZBIN-based inspection and refinement of three automatically generated MGS.** **(A–C)** Contigs ≥1,000 nt and cut into fragments of 5,000 nt in length (see “Materials and Methods”) are shown. Symbols highlighted in red represent fragments coding for at least one gene included in the respective MGS, blue symbols represent fragments not encoding any gene in the respective MGS. Red polygons indicate the selection boundaries manually defined in VIZBIN. **(A)** MGS00153 – Alphaproteobacteria-like. **(B)** MGS00248 – Mollicutes-like. **(C)** MGS00113 – Cyanobacteria-like. Red circles indicate two additional clusters represented by the MGS that were chosen for further inspection.

In order to prioritize the automatically generated clusters for inspection and refinement using VIZBIN, all 365 MGS were taxonomically classified using PHYLOPHLAN. This allowed us to focus our efforts on the recovery of genomic complements derived from hitherto undescribed organisms. Among the 365 MGS, 16 lacked a taxonomic assignment at the order/class-level or lower (**Figure 2**). Three of these 16 MGS were selected for further processing using VIZBIN: MGS00153 – an Alphaproteobacteria-like MGS, MGS00248 – a Mollicutes-like MGS, and MGS113 – a Cyanobacteria-like MGS. For each of the three MGS, the sample that exhibited the highest average fold-coverage of the respective MGS was chosen and the contigs of that sample were used as input for VIZBIN-based visualization. Using VIZBIN, contigs were highlighted, which contained genes of the MGS of interest, and the contig bins were refined by manual selection (**Figure 3**).

**FIGURE 4:**
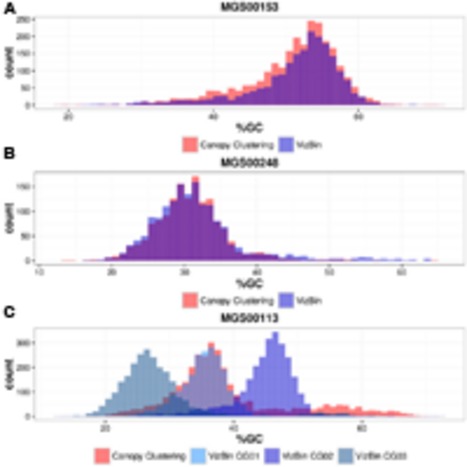
**Gene-wise %GC distributions of automatically generated vs. manually defined sequence clusters.** **(A)** MGS00153. **(B)** MGS00248. **(C)** MGS00113.

Contigs encoding genes of MGS00153 and MGS00248 were mostly found within a single cluster each with few outlying contigs in the respective VIZBIN maps (**Figures 3A,B**). Moreover, the gene-wise %GC distributions of the automatically generated or manually defined clusters were highly similar for these two MGS (**Figures 4A,B**). In contrast to MGS00153 and MGS00248, the genes of the Cyanobacteria-like MGS00113 were present in contigs forming part of multiple contig clusters in the VIZBIN map (**Figure 3C**). The gene-wise %GC distribution of MGS00113 exhibited a large spread as well as relatively high frequencies of genes with divergent %GC, in particular for high %GC values (**Figure 4C**). This further supported that MGS00113 represented a mixture of population-level genomic complements. The three most prominent clusters (composite genomes – CGs) representing genes of MGS00113 were chosen in the VIZBIN map for further inspection and are referred to as MGS00113-CG01, MGS00113-CG02, and MGS00113-CG03 in the following (**Figure 3C**). All CGs were found to be almost complete with only MGS00113-CG03 containing several essential genes in multiple copies (65/107). An online BLAST search of the CGs’ *rpoB* genes revealed limited sequence similarity for MGS00113-CG01 (78% identity over 11% of the query sequence against *Clostridium saccharobutylicum* DSM 13864; top hit), while MGS00113-CG02 and MGS00113-CG03 showed higher sequence similarities (77% identity over 93% of the query sequence against *Coprococcus* sp. ART55/1 and 75% identity over 92% of the query sequence against *Clostridium botulinum* A str. Hall, respectively; top hits). Given the PHYLOPHLAN-based Cyanobacteria-like classification of MGS00113, CG01 was compared separately to genomes derived from representatives of the recently defined lineage of GIT-borne Cyanobacteria-like microorganisms, the Melainabacteria (Di Rienzi et al., 2013; Soo et al., 2014). This comparison revealed high sequence similarity with MEL.B1 (mean ANI of 97.11%, **Supplementary Figure S3**) and suggested that these two (MGS00113-CG01 and MEL.B1) represent closely related strains. MGS00113-CG01 and MGS00113-CG03 were omitted from further analysis due to high sequence similarity to the existing MEL.B1 genome or due to a high degree of contamination, respectively.

While the automatically generated and the manually defined clusters were largely concordant with respect to MGS00153 and MGS00248, thus lending mutual support, the case of MGS00113 demonstrated the importance of *post hoc* inspection and refinement. In particular, the PHYLOPHLAN-based taxonomic classification of MGS00113 suggested the sequences to be derived from a cyanobacterial organism. However, this classification was misleading as MGS00113 represented a mixture of genomic fragments of at least two classes and at least three distinct organisms.

### Targeted Recovery of Genomic Complements Derived from Cyanobacteria-Like Organisms

Motivated by the high similarity between MGS00113-CG01 and MEL.B1, it was intriguing to assess whether further hitherto undescribed Cyanobacteria-like genomic complements could be recovered from the MuSt metagenomic data via *de novo* application of VIZBIN. To this end, Cyanobacteria-like sequences (“beacon contigs”) were used to highlight candidate cyanobacterial clusters and three population-level genomes were subsequently recovered.

#### Identification of Cyanobacteria-Like Sequence Groups

Phylogenetic marker genes encoded by the *de novo* assembled contigs from the MuSt samples were first identified and taxonomically classified using AMPHORA2. The phylogenetic marker gene which was annotated to belong to the Cyanobacteria phylum most often was the gene encoding the DNA-directed RNA polymerase subunit beta (*rpoB*). A total of 139 cyanobacterial copies of this gene were found in 42 of the 55 MuSt samples. Subsequent sequence similarity-based clustering of complete genes resulted in three sequence clusters which are referred to herein as “CLSGs” and the respective representative genes are referred to as “CLSG marker genes” (**Figure 1**).

**FIGURE 5:**
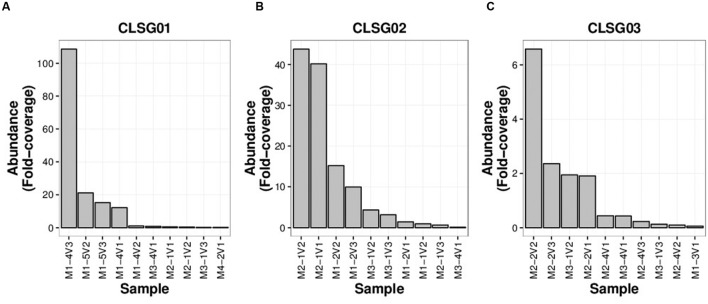
**CLSG marker gene rank-abundance plots for the ten samples with the highest apparent abundance of the respective populations.** **(A)** CLSG01. **(B)** CLSG02. **(C)** CLSG03. The fold-coverage of CLSG genes was used as a proxy for the abundance of the respective CLSG. Samples are denoted as illustrated by the following example – M1-4V3: Family 1, individual 4 of that family, sample 3 of that individual.

Fold-coverages of the CLSG marker genes were used as proxies for estimating population sizes to select for the sample with the highest fold-coverage for each CLSG. The CLSGs were numbered based on descending fold-coverage values with CLSG01 exhibiting the highest fold-coverage in any sample (≈ 109 fold-coverage; M1-4V3, i.e., family 1, individual 4 of that family, sample 3 of that individual), CLSG02 was found to have a fold-coverage of ≈ 44 (M2-1V2), and CLSG03 was found to exhibit a quite low fold-coverage (≈ 6.6-fold-coverage; M2-2V2; **Figure 5**). Pronounced intraindividual variations over time for each of the three CLSGs were observed, representing up to two orders of magnitude of differences for CLSG01 (M1-4V2 vs. M1-4V3, i.e., samples at timepoints 2 and 3 of the same individual, in **Figure 5A**).

**FIGURE 6:**
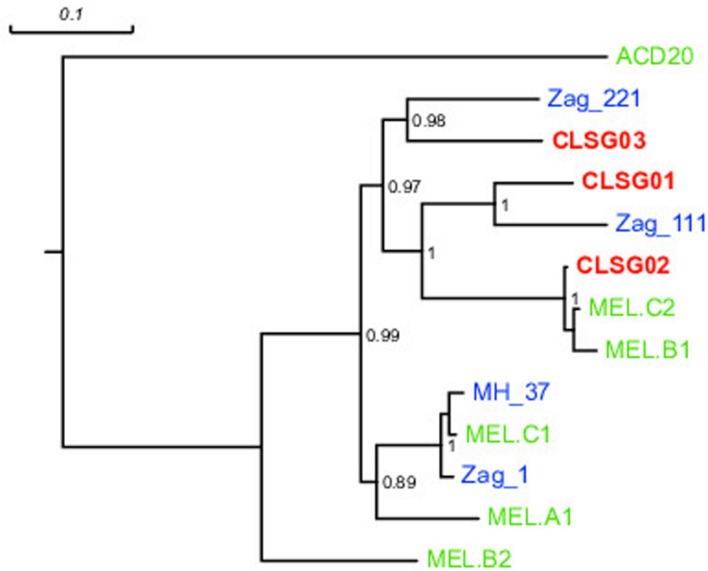
***rpoB* gene-based maximum likelihood phylogenetic tree of previously published Melainabacteria genomes and herein recovered CLSG genomes.** Green, blue, and bold red text represent sequences derived from genomes recovered in Di Rienzi et al. (2013) and Soo et al. (2014), and herein, respectively. The environment-derived Melainabacterium ACD20 was chosen as outgroup. Substitutions per site are indicated by the scale-bar on top. Branch support values ≥0.5 are shown for the respective splits.

In order to compare our CLSG populations to those previously reported in samples from other geographical locations (Di Rienzi et al., 2013) as well as other hosts (Soo et al., 2014), a phylogenetic tree based on the three herein identified CLSG *rpoB* genes and *rpoB* genes of 10 previously characterized Melainabacteria population-level genomes was constructed (**Figure 6**). Inspection of the tree revealed CLSG02 to be closely related to the MEL.B1 and MEL.C2 genomes. CLSG01 and CLSG03, however, exhibited far lower sequence similarity to previously characterized Melainabacteria and appear to be more distantly related. The environmental Melainabacteria population (ACD20) was found to be basal to the GIT-derived populations. None of the three CLSGs represented outgroups but rather shared phylogenetic relationships with the GIT-derived Melainabacteria.

Possible alternatives to the *rpoB* gene-based approach applied here were the use of *gyrA* or *gyrB* (Kasai et al., 2000; Aranaz et al., 2003; Baker et al., 2004; Menard et al., 2016), or the use of a gene set, e.g., as annotated by AMPHORA2. However, *gyrA* and *gyrB* seemed to be especially promising for the separation of particularly closely related organisms and the use of single marker genes provided important advantages in terms of simplicity over more complex marker gene sets.

#### Recovery of Population-Level Genomes Guided by CLSG Marker Genes

**FIGURE 7:**
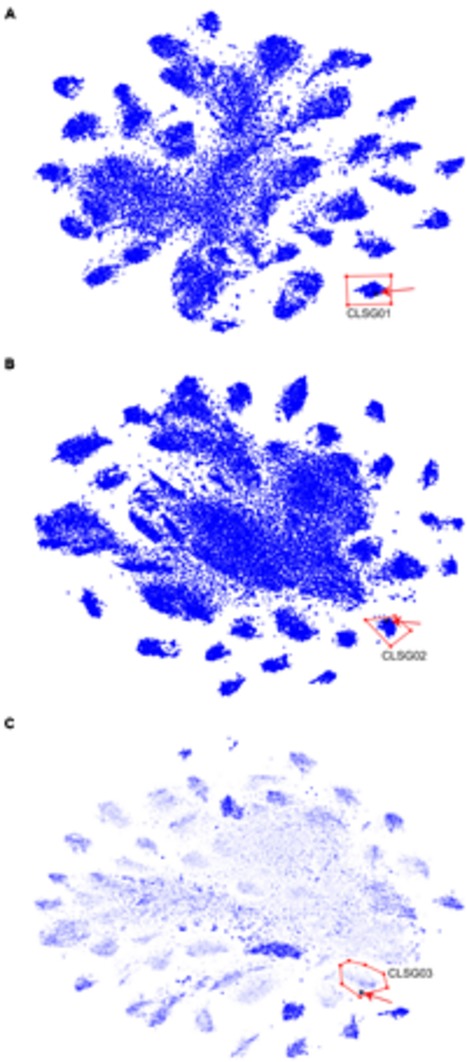
**Visualization and cluster selection of Cyanobacteria-like population-level genomes in VIZBIN.** **(A)** CLSG01. **(B)** CLSG02. **(C)** CLSG03. **(A–C)** Original contig sequences ≥1,000 nt, cut into fragments of 5,000 nt. Blue points represent sequences from the samples’ metagenomic assemblies. The black star-like shape represents the beacon contig encoding the CLSG marker gene and is highlighted by a red arrow for quicker detection. Red polygons delineate the selected sequence clusters. **(C)** The opaqueness is proportional to the natural logarithm of the fold-coverage and is used here to improve cluster boundary detection.

For each of the three CLSGs, the sample with the highest fold-coverage of the respective CLSG marker genes was selected and visualized using VIZBIN for cluster definition (**Figure 7**). The clusters containing the CLSG01 and CLSG02 beacon contigs were peripheral and well separated (**Figures 7A,B**). In addition, for CLSG03, the fold-coverage was added as an opaqueness value of the points to improve the delineation of cluster boundaries, thereby facilitating cluster selection (**Figure 7C**). The recovered population-level genomes are herein referred to as CLSG genomes and an overview of genomic and functional features is provided in **Table 1.**

**Table 1:** Genomic and functional features of refined and re-assembled population-level genomes.

Population-level genome	MGS00153	MGS00248	MGS00113-CG02	CLSG01	CLSG02	CLSG03
Originating sample	M2-3V2	M2-1V2	M2-1V1	M1-4V3	M2-1V2	M2-2V2
Size (bp)	1,954,779	1,555,611	2,970,300	1,871,540	2,180,307	1,916,257
# Contigs	157	113	408	47	83	551
%GC	50.81	30.48	44.49	32.25	35.21	35.98
# CDS	2,049	1,410	2,605	1,848	2,139	1,876
# Protein-coding CDS	2,006	1,371	2,560	1,809	2,095	1,852
# rRNAs (complete or partial)	2x 16S	0	4x 16S/23S	0	5S/23S	0
# tRNAs	41	39	40	39	42	24
tRNAs missing for ^†^	I/F	F/N	I/F/Y	none	none	C/D/F/H/N/T/Y
# Essential genes (out of 107)	105	76	102	106	106	81
# Multi-copy essent. genes	1	1	17	3	3	3
EMP pathway complete ^‡^	Yes	No	Yes	Yes	Yes	Yes
PP pathway complete ^∗^	No	No	No	No	No	No
TCA cycle complete	No	No	No	No	No	No
Entner–Doudoroff pathway complete	No	No	No	No	No	No
Predicted fermentation products ^§^	ET/AC	ET/FO/AC	ET/LA/FO/AC	ET	ET/LA/FO	ET
Classical electron transport chain	No	No	No	No	Partial	No
Rnf electron transport complex	Yes	Partial	Partial	No	No	No
ATP synthase	Yes	Yes	Yes	Yes	Yes	Yes
# Flagellar genes	4	0	42	15	54	12
Vitamins: B1/B2/B3/B6/B9/B12/H ^∇^	-/-/-/-/+/-/-	-/-/-/-/-/-/-	-/+/+/-(?)/+/-(?)/-	-/+/-/-/(?)/-/+	-/+/-/-/-(?)/-/+	-/+/-/-/-/-/+


*^†^: C, cysteine; D, aspartic acid; F, phenylalanine; H, histidine; I, isoleucine; N, asparagine; T, threonine; Y, tyrosine. ^‡^: EMP, Embden-Meyerhof-Parnas. ^∗^: PP, pentose phosphate. ^¶^: TCA, tricarboxylic acid. ^§^ : ET, ethanol; AC, acetate; LA, lactate; FO, formate. ^∇^: -, incomplete; +, complete; ?, partially complete.*

**FIGURE 8:**
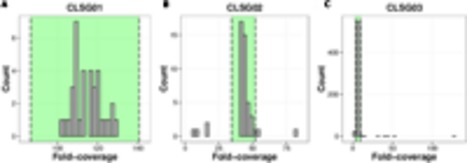
**Coverage distributions of individual CLSG genomes.** **(A)** CLSG01 genome. **(B)** CLSG02 genome. **(C)** CLSG03 genome. The distributions of the average per-contig fold-coverages are depicted. Contigs with average fold-coverage values within the range highlighted in green between the two dashed lines are preserved. Contigs outside of this range exhibited exceptionally high or low fold-coverage values and were discarded, i.e., the absolute value of the modified *Z*-score was greater than 3.5.

The CLSG01 and CLSG02 genomes both exhibited a high degree of completeness (106/107 essential genes) and a low degree of contamination (3/107 essential genes in multiple copies; **Table 1**). In contrast, the CLSG03 genome was less complete (81/107) and more contaminated (11/107 in multiple copies). The fold-coverage distributions of contigs of the individual CLSG genomes indicated that only few contigs exhibited divergent fold-coverage values, i.e., they were of exceptionally high or low fold-coverage (**Figure 8**). This supported the low degree of contamination as already indicated by the essential genes’ abundance patterns. The CLSG03 genome exhibited a relatively low overall fold-coverage (<10×). Accordingly, the reduced degree of completeness for this CLSG genome could be due to insufficient sequencing depth for the corresponding population and thus suboptimal assembly of the population-level genome. In contrast, the almost completely recovered genome of the most abundant CLSG, CLSG01, accounted for around 5% of the reads in the originating sample (M1-4V3), thus constituting a sizeable fraction of the sample’s metagenomic complement.

### Taxonomic and Functional Characterizations of Recovered Genomes

We expected that all genomic complements from a single microbial population would exhibit similar fold-coverages in a given sample. Accordingly, the manually recovered genomic complements were refined by discarding contigs with extreme fold-coverages (**Figure 8**) and the reads constituting the preserved contigs were re-assembled (**Figure 1**). The refined and re-assembled population-level genomes were characterized taxonomically as well as functionally. The main results are summarized here with further details provided in the Supplementary Notes.

#### MGS00153 – Alphaproteobacteria-Like Population

**FIGURE 9:**
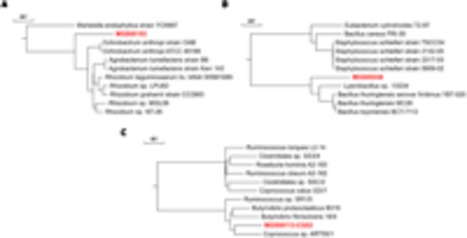
***rpoB* gene-based phylogenetic trees for refined MGS genomes and their ten nearest neighbors according to MOLE-BLAST.** **(A)** MGS00153 – Alphaproteobacteria-like. **(B)** MGS00248 – Mollicutes-like. **(C)** MGS00113-CG02 – Clostridiales-like. Bold, red text represents the sequences derived from population-level genomes recovered in this work. Scale bars on top represent substitutions per site.

The refined and re-assembled MGS00153 genome is likely derived from a member of the Alphaproteobacteria class (**Figure 2**; **Supplementary Table S2**). Unambiguous assignment to a lower taxonomic level, e.g., order or family, remained unresolved: two partially recovered 16S ribosomal RNA (rRNA) genes suggested a placement within the Kopriimonadales order while a MOLE-BLAST search of the recovered *rpoB* gene suggested the Rhizobiales to be the most closely related order (**Figure 9A**). This ambiguity could be due to the lack of reference sequences derived from more closely related microorganisms.

Functional characterization of the MGS00153 genome suggested that the identified organism has a fermentative lifestyle with ethanol and acetate as fermentation products, is auxotroph for most of the vitamins considered, and is unflagellated (**Table 1**; Supplementary Material).

#### MGS00248 – Mollicutes-Like Population

The Mollicutes class has been assigned to the Firmicutes phylum (Johansson and Pettersson, 2002) and has subsequently been reassigned to the Tenericutes phylum (Ludwig et al., 2009), an ambiguity which is reflected in the case of MGS00248 (**Figures 2** and **9B**, **Supplementary Table 3**, **Supplementary Figure S4B**). The genome of MGS00248 exhibited features which are typical for representatives of the Mollicutes class, e.g., genome size (1.5 Mbp), and lack of flagellar assembly genes (**Table 1**). Based on these inferred physiological traits, the organism is suggested herein to be derived from a member of the Mollicutes class.

#### MGS00113-CG02 – Clostridiales-Like Population

The organism represented by MGS00113-CG02 is likely a member of the Clostridiales order based on the taxonomic analysis results (**Supplementary Table S4**, **Supplementary Figure S4C**, **Figure 9C**). Furthermore, it is suggested to be a member of a butyrate-producing subgroup of organisms from the Lachnospiraceae family within the Clostridiales order as the MGS00113-CG02 genome was found to encode a butyrate-kinase (fig| 6666666.163361.peg.1219; Meehan and Beiko, 2014).

#### Cyanobacteria-Like Populations

CLSG02 and MEL.B1 shared a high sequence similarity (mean ANI of 97.03%, **Supplementary Figure S6**; **Figure 6**) and thus likely represent the same species. Moreover, MGS00113-CG01 and CLSG02 were found to be almost identical (mean ANI of 100%, **Supplementary Figure S7**). In contrast, the CLSG01 and CLSG03 genomes were more distantly related to previously recovered genomes from the Melainabacteria class (**Figure 6**; mean ANI of 77.63 and 77.77% to their respective closest relatives, **Supplementary Figures S8** and **S9**) and thus constitute novel melainabacterial representatives.

**FIGURE 10:**
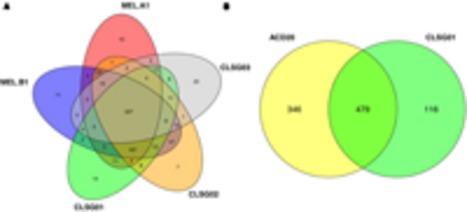
**Comparisons of SEED-based functional roles of previously recovered Melainabacteria genomes and CLSG genomes recovered herein.** **(A)** The overlaps of SEED-based functional roles for two previously recovered, GIT-derived Melainabacteria population-level genomes (MEL.A1 and MEL.B1; Di Rienzi et al., 2013) and the three CLSG population-level genomes recovered in this work (CLSG01–03) are shown. **(B)** The overlap of SEED-based functional roles between a previously recovered groundwater-derived (ACD20) Melainabacteria population-level genome (Di Rienzi et al., 2013) and the CLSG01 genome is shown.

Large overlaps in the functional potential with other GIT-derived Melainabacteria and limited overlap with an environment-derived Melainabacterium corroborated the taxonomic assignment of the CLSG genomes to the GIT-derived Melainabacteria class within the Cyanobacteria phylum (**Figure 10**; **Supplementary Tables S5**–**S7**). Most notably, no photosynthesis genes were found, the genomes were predicted to encode genes for vitamin B production (B_2_, B_9_, H), and represent obligate anaerobic fermenters (**Table 1**; Supplementary Material). While CLSG02 and MEL.B1 likely represent the same species, genome-specific functions were identified, e.g., a HigB/HigA toxin-antitoxin (TA) system (Christensen-Dalsgaard et al., 2010) was found to be encoded by the CLSG02 genome, yet, this is not encoded by the other melainabacterial genomes.

## Conclusion

The concurrent characterization of community composition and functional potential through metagenomic sequencing is of great importance for the analysis of microbial communities. However, despite sequence assembly, metagenomic data typically remains fragmented which in turn hampers population-level analyses. Moreover, several microbial lineages are underrepresented in current reference genome catalogs. Therefore, reference-independent computational binning approaches are required for the deconvolution of metagenomic data into population-level genomes derived from hitherto undescribed microorganisms. Here, we applied two reference-independent binning approaches for the identification, recovery, and refinement of such genomes derived from the human GIT. The expansion of the repertoire of currently available reference genomes by the herein recovered representatives is expected to benefit human microbiome-based studies, eventually resulting in improved taxonomic profiling and functional characterization.

First, a multi sample-based, automated binning approach, CANOPY CLUSTERING, was used to perform an initial binning, which was followed by taxonomic classification of the automatically generated bins. The taxonomic classification enabled us to place a focus on sequence clusters likely derived from hitherto undescribed microbial populations for VIZBIN-based *post hoc* inspection and refinement. The importance of complementary approaches, such as VIZBIN, that enable human scrutiny of automatically generated sequence clusters is in particular highlighted by the required refinement of MGS00113 (multiple apparent clusters in the VIZBIN map, large spread in %GC of the gene content). Overall, the combination of the two binning approaches resulted in the recovery of one population-level genome from the Alphaproteobacteria class (MGS00153), one from the Mollicutes class (MGS00248), and one from the Clostridiales order (MGS00113-CG02).

Second, a targeted *de novo* recovery of population-level genomic complements from the Melainabacteria was performed, resulting in the recovery of two almost complete genomes and one partial genome (CLSG01–03). The assignment to the Melainabacteria was supported by phylogenetic, genomic, and functional analyses. While large fractions of the functional potential are shared between the herein recovered and the previously described melainabacterial genomes, individual genomes were found to encode genome-specific functions. Moreover, pronounced intraindividual population-abundance variations were observed over time which included differences in estimated population sizes spanning two orders of magnitude.

The observed intraindividual variations of population-abundances highlight the importance of longitudinal studies in the context of *in situ* genome recovery. Despite extensive efforts toward the recovery of microbial genomes from the human GIT, several hitherto undescribed GIT-derived population-level genomes were recovered in this work using the complementary combination of an automated and a user-driven binning approach. It is thus suggested that automated binning approaches should be supplemented with user-driven approaches to ensure the recovery of high-quality population-level genomes from longitudinally collected metagenomic data.

## Author Contributions

CdB and PW conceived the study, participated in its design and coordination, and drafted the manuscript. CL carried out the binning, performed the taxonomic and phylogenetic analyses, and wrote the paper. EM performed the functional analyses of the recovered and contributed to writing the paper. AH-B carried out the biomolecular extractions and contributed to writing the paper. MH participated in the generation of the functional annotations. LL participated in the biomolecular extraction. AH participated in the design of the study and in the sample and data collection. PM carried out the sequence assembly and gene prediction. CL, EM, AH-B, and PW interpreted the data. All authors read and approved the final manuscript.

## Conflict of Interest Statement

The authors declare that the research was conducted in the absence of any commercial or financial relationships that could be construed as a potential conflict of interest.

## Footnotes

1 https://github.com/MadsAlbertsen/multi-metagenome/blob/master/R.data.generation/essential.hmm

2 http://enve-omics.ce.gatech.edu/ani/

3 http://blast.ncbi.nlm.nih.gov/moleblast/moleblast.cgi

4 http://ggkbase.berkeley.edu/mel/organisms.

5 http://www.phylogeny.fr/simple_phylogeny.cgi

6 http://broadinstitute.github.io/picard

7 http://rast.nmpdr.org/
