# Supplementary material for: Identification, Recovery, and Refinement of Hitherto Undescribed Population-Level Genomes from the Human Gastrointestinal Tract
Source: Front Microbiol. 2016 Jun 21;7:884. doi: 10.3389/fmicb.2016.00884 (PMC4914512; doi:10.3389/fmicb.2016.00884)
Supplement: Supplementary file 1 [file Presentation_1.PDF]

Supplementary Material:

Identification, recovery, and refinement of hitherto undescribed population-level genomes from the human gastrointestinal tract

Cedric C Laczny [1,§], Emilie EL Muller [1], Anna Heintz-Buschart [1], Malte Herold [1], Laura A Lebrun [1], Angela Hogan [2], Patrick May [1], Carine de Beaufort [1,3], and Paul Wilmes [1,*]
*Correspondence:
Paul Wilmes
paul.wilmes@uni.lu

1 SUPPLEMENTARY NOTES – TAXONOMIC & FUNCTIONAL DESCRIPTIONS

MGS00153 – Alphaproteobacteria-like population

AMPHORA2 analysis suggested that the refined and reassembled metagenomic species (MGS)00153 genome is likely derived from a member of the Alphaproteobacteria class (**Supplementary Table 2**), thereby supporting the PhyloPhlAn-based taxonomic classification (**Figure 2**). For 112 of 157 contigs, no significant alignment could be found by a BLAST search followed by the lowest common ancestor (LCA)-option in MEGAN (**Supplementary Figure 4(A)**). This suggested that not a single genome of a closely related organism existed in the National Center for Biotechnology Information (NCBI) non-redundant nucleotide database. Two partial genes (167 bp) encoding the 16S ribosomal RNA (rRNA) were found in the reconstructed genome. The absence of the complete ribosomal operon is not unexpected, as in general, *de novo* assembly of this operon is limited (Miller et al., 2011). Moreover, genes derived from this operon exhibit distinct genomic signatures (Laczny et al., 2014). These distinct signatures challenge sequence fragment recovery using VizBin if no or too little flanking sequences representative of the originating genome's signature are present. A BLAST search of the recovered partial rRNA gene revealed 81% identity with an Alphaproteobacterium of the Kopriimonadales order. In addition to the partially recovered rRNA gene, a complete *rpoB* gene was recovered (fig|6666666.163363.peg.142). Based on a MOLE-BLAST search of the recovered *rpoB* gene, MGS00153 appeared to be most closely, yet distantly related to members of the Rhizobiales order and formed a distinct group (**Figure 9(A)**).

While all genes required for a complete ATP synthase assembly were recovered for the refined and reassembled population-level genome of MGS00153, the other complexes of the classical electron transport chain were missing, indicating that the ATPase may rather be involved in ATP hydrolysis to generate membrane potential (**Table 1**; Supplementary Material online). The Embden-Meyerhof-Parnas (EMP) pathway was found to be complete, yet other pathways of the central carbon metabolism were found to be incomplete. A sugar transporter was also found to be encoded by the recovered genome. Furthermore, MGS00153 was predicted to produce ethanol and acetate as fermentation products. These results suggested

that the identified organism has a fermentative lifestyle. However, the genome was found to encode a recently discovered ion-motive electron transport complex, Rnf, which energetically couples cellular ferredoxin to the pyridine nucleotide pool (Biegel et al., 2011), as well as potential ferredoxin-reducing enzymes, e.g., Pyruvate:ferrodoxin/flavodoxin reductase EC: 1.2.7.- – fig|6666666.163363.peg.742. The Rnf complex and the ATPase may thus be involved in chemiosmotic ATP synthesis (Biegel et al., 2011). The genome lacked a gene for the Isoleucine and Phenylalanine tRNAs and the organism was predicted to be auxotroph for most of the vitamins analysed (**Table 1**; Supplementary Material online). The organism is likely to be unflagellated as only a very limited set of genes associated with flagellar assembly were found.

MGS00248 – Mollicutes-like population

While PHYLOPHLAN classified MGS00248 as a member of the Mollicutes class within the Tenericutes phylum (**Figure 2**), only about half of the marker genes used by AMPHORA2 and present in MGS00248 were annotated to belong to the Mollicutes (**Supplementary Table 3**). The remaining marker genes encoded by the recovered MGS00248 genome were mostly classified to be derived from members of the Erysipelotrichia class within the Firmicutes phylum. The BLAST-search of the refined and reassembled MGS00248 contigs revealed 45 Firmicutes-like contigs and 10 Tenericutes-like contigs (**Supplementary Figure 4(B)**). A total of 36 contigs were not assigned to a bacterial taxon or did not result in significant alignments against the NCBI's "nr/nt" database. No gene encoding a rRNA was found for MGS00248. However, one complete and one partial *rpoB* gene were annotated and the sequence of the complete *rpoB* gene (fig|6666666.163364.peg.393) was used to construct a phylogenetic tree via MOLE-BLAST (**Figure 9(B)**). This would suggest a phylogenetic placement in the Firmicutes phylum rather than the Tenericutes phylum. However, the Mollicutes class has been assigned to the Firmicutes phylum (Johansson and Pettersson, 2002) and has subsequently been reassigned to the Tenericutes phylum (Ludwig et al., 2009). This ambiguity in the taxonomic assignments appears to be reflected in the case of MGS00248.

With 1.5 Mbp, about 1,400 genes, a rather low %GC and truncated metabolic pathways, this population-level genome exhibited features typical for representatives of the Mollicutes class. Although a large number and variety of sugar transporters were found, the pyruvate kinase allowing the final substrate-level phosphorylation during glycolysis was missing from the EMP pathway. However, the gene encoding the enzyme to perform the reverse reaction (Pyruvate-phosphate dikinase EC: 2.7.9.1 – fig|6666666.163364.peg.442) was found, suggesting that this organism is potentially performing gluconeogenesis. Similarly to the MGS00153, MGS00248 was predicted to encode for an Rnf complex and an ATP synthase. Furthermore, the respective organism is likely to be unflagellated and auxotroph for most of the vitamins (**Table 1**).

MGS00113-CG02 – Clostridiales-like population

MGS00113-CG02 is likely a member of the Clostridiales order based on the AMPHORA2 results (**Supplementary Table 4**). While 206 Firmicutes-like contigs were identified, 195 contigs remained unclassified by an online BLAST search (**Supplementary Figure 4(C)**). No complete 16S rRNA gene was recovered, but a BLAST search of the recovered partial sequences supported the assignment to the Clostridia class. *rpoB* gene-based phylogenetic analysis revealed *Coprococcus* sp. ART55 1 to be the closest, yet distant, representative (**Figure 9(C)**).

The refined and reassembled genome of MGS00113-CG02 was found to encode all genes of the EMP pathway while the pentose phospate (PP) and tricarboxylic acid (TCA) pathways were both incomplete (Supplementary Material online). The organism is predicted to be able to produce various fermentation

products (ethanol, lactate, acetate, formate) from diverse substrates including, e.g., cellobiose, arbutin, or salicin. Moreover, the recovered MGS00113-CG02 genome was found to encode a butyrate kinase (fig|6666666.163361.peg.1219). This suggested that MGS00113-CG02 may be a member of a subgroup of organisms from the Lachnospiraceae family which also encode this gene, along with *Coprococcus comes* ATCC 27758 and *Coprococcus eutactus* ATCC 27759 (Meehan and Beiko, 2014). The prevalence of gastrointestinal tract (GIT)-borne butyrate-producing bacteria has previously been linked to host health (Canani et al., 2011; Yadav et al., 2013; Chang et al., 2014), for example in relation to its immunomodulatory potential (Zimmerman et al., 2012; Atarashi et al., 2013; Furusawa et al., 2013).

Furthermore, analyses of the refined and reassembled genome revealed that the respective organism is likely to be flagellated, and the genome encodes an ATP synthase as well as genes of the Rnf complex, and several complete or nearly complete pathways for vitamin biosynthesis (**Table 1**).

Cyanobacteria-like populations

As expected, the AMPHORA2 results suggested that the recovered and reassembled population-level genomes (Cyanobacteria-like sequence group (CLSG)01 – 03) are likely derived from organisms of the Cyanobacteria phylum (**Supplementary Tables 5 – 7**). The majority of the contigs remained unclassified by the BLAST + MEGAN-based analyses of all of the three genomes (**Supplementary Figure 5**). For the CLSG02 genome, a pairwise comparison to the recovered Melainabacteria population-level genome MEL.B1 resulted in a mean average nucleotide identity (ANI) of 97.03% (**Supplementary Figure 6**) thus supporting the close phylogenetic relationship of these two genomes as already indicated by the *rpoB*-based phylogenetic tree (**Figure 6**). Therefore, MEL.B1 and CLSG02 most likely represent the same species. Moreover, MGS00113-CG01 and CLSG02 were found to be almost identical (ANI of 100%, **Supplementary Figure 7**). CLSG01 and CLSG03 were however more distantly related to previously recovered genomes from the Melainabacteria class (**Figure 6**; mean ANI of 77.63% and 77.77% to their respective closest relatives, **Supplementary Figures 8 – 9**) and thus constitute novel melainabacterial representatives.

In addition to the phylogenetic and genomic analyses of the herein recovered and reassembled CLSG genomes, their functional potential, as characterised by SEED subsystems roles, was analysed and compared to the re-analysed functional potential of a subset of previously characterised genomes of the Melainabacteria class (Di Rienzi et al., 2013). The subset consisted of MEL.A1 and MEL.B1 as well as ACD20 which were chosen due to the good assembly qualities of the first two and the latter to serve as outgroup. The groundwater-derived Melainabacteria genome (ACD20) comprised a larger number of SEED subsystems roles (825) compared to the GIT-derived Melainabacteria or CLSG genomes (e.g., 649 for CLSG02) (**Figure 10**). Large overlaps in roles were observed between MEL.A1, MEL.B1, and CLSG01 – 03 (**Figure 10(A)**). The most complete CLSG (CLSG01) was compared to the groundwater-derived melainabacterial genome (ACD20) and the functional overlap was more limited (**Figure 10(B)**).

Despite their close phylogenetic relationship, high genomic sequence similarity, as well as a large overlap in the functional potentials of CLSG02 and MEL.B1, genome-specific functions were identified (**Figure 10(A)**). Notably, CLSG02, but not MEL.B1, was found to encode a HigB/HigA toxin-antitoxin (TA) system consisting of the HigB toxin protein and its associated HigA antitoxin protein (Christensen-Dalsgaard et al., 2010). TA systems are associated with programmed cell death and have been found in numerous bacteria (Pandey and Gerdes, 2005; Fozo et al., 2010; Yamaguchi et al., 2011). TA systems were originally discovered on plasmids and have since been found to also be present on chromosomes (Hayes, 2003). Plasmid-encoded TA systems are assumed to eliminate plasmid-free cells while chromosome-encoded TA

systems supposedly induce reversible cell cycle arrest or programmed cell death under stress conditions (Hayes, 2003). However, melainabacterial genomes have not been reported to contain TA systems and no such system was found in the genomes of MEL.A1, MEL.B1, or ACD20. It thus may represent a genomic element recently acquired by CLSG02, e.g., via horizontal gene transfer.

The genomes of CLSG01 and CLSG02 were both found to contain tRNAs for all the 20 typical amino acids, while the tRNAs for asparagine, aspartic acid, cysteine, histidine, phenylalanine, threonine, and tyrosine were not recovered for CLSG03 (**Table 1**). Analyses of the energy metabolism revealed that the refined CLSG genomes encode a complete EMP pathway, and incomplete PP and TCA pathways, similar to the previously described genomes of MEL.A1 and MEL.B1 (Di Rienzi et al., 2013; Soo et al., 2014). The organisms represented by CLSG01 – 03 were also predicted to be obligate anaerobic fermenters and were found to contain fermentative and degradative enzymes for the production of ethanol and formate, in line with previous reports (Di Rienzi et al., 2013; Soo et al., 2014). Various saccharide-related transporters were recovered, e.g., maltose transporters, multiple sugar transporters as well as cellobiose phosphotransferases, some of which were also previously described, thus highlighting sugar acquisition by the organisms represented by CLSG01 – 03 (Di Rienzi et al., 2013; Soo et al., 2014). CLSG02 was found to encode genes related to the electron transport chain complex I (e.g., *nuoA*, *nuoB*) although none of the bacterial complexes (I,II, and IV) were found to be complete, as originally described for MEL.B1 (Di Rienzi et al., 2013). In contrast, all genes required for a complete ATP synthase assembly were recovered. This could, even in the absence of a complete electron transport chain, allow production of ATP using a proton translocation mechanism as genes for membrane energisation, e.g., a pyrophosphate-energised proton pump, were found (Di Rienzi et al., 2013). MEL.B1 was previously reported to likely be flagellated which is reflected in a large number of genes associated with flagellar assembly (Di Rienzi et al., 2013). While a high number of flagellar assembly genes was found for CLSG02, CLSG01 and CLSG03 exhibited only a small number of related genes (**Table 1**). No photosynthesis genes were identified (Supplementary Material online), as it is expected for members of the Melainabacteria (Di Rienzi et al., 2013; Soo et al., 2014). Furthermore, Melainabacteria are predicted to produce B vitamins (Di Rienzi et al., 2013) and all CLSG genomes in this work were predicted to be capable of vitamin B production (B_2, B_9, H; **Table 1**).

Albeit distinct (except CLSG02), the refined CLSG genomes bared a close resemblance to previously characterised Melainabacteria genomes with respect to their encoded functional complement. This corroborated the assignment to the Melainabacteria class which is hereby extended by hitherto undescribed representatives.

2 SUPPLEMENTARY FIGURES

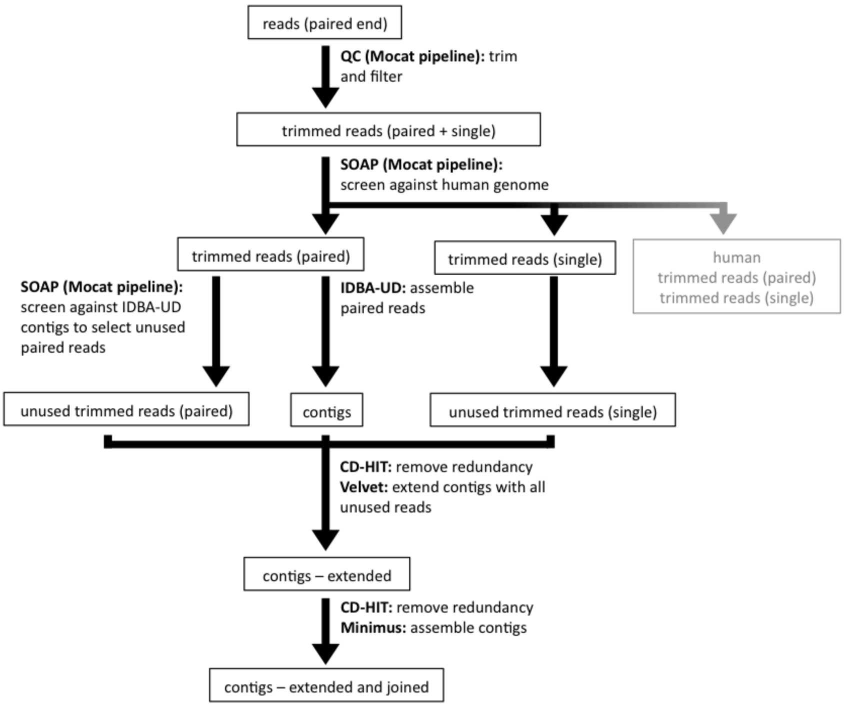

Supplementary Figure 1. Metagenomic assembly workflow. Details about software versions and parameters are provided in the Material and Methods section.

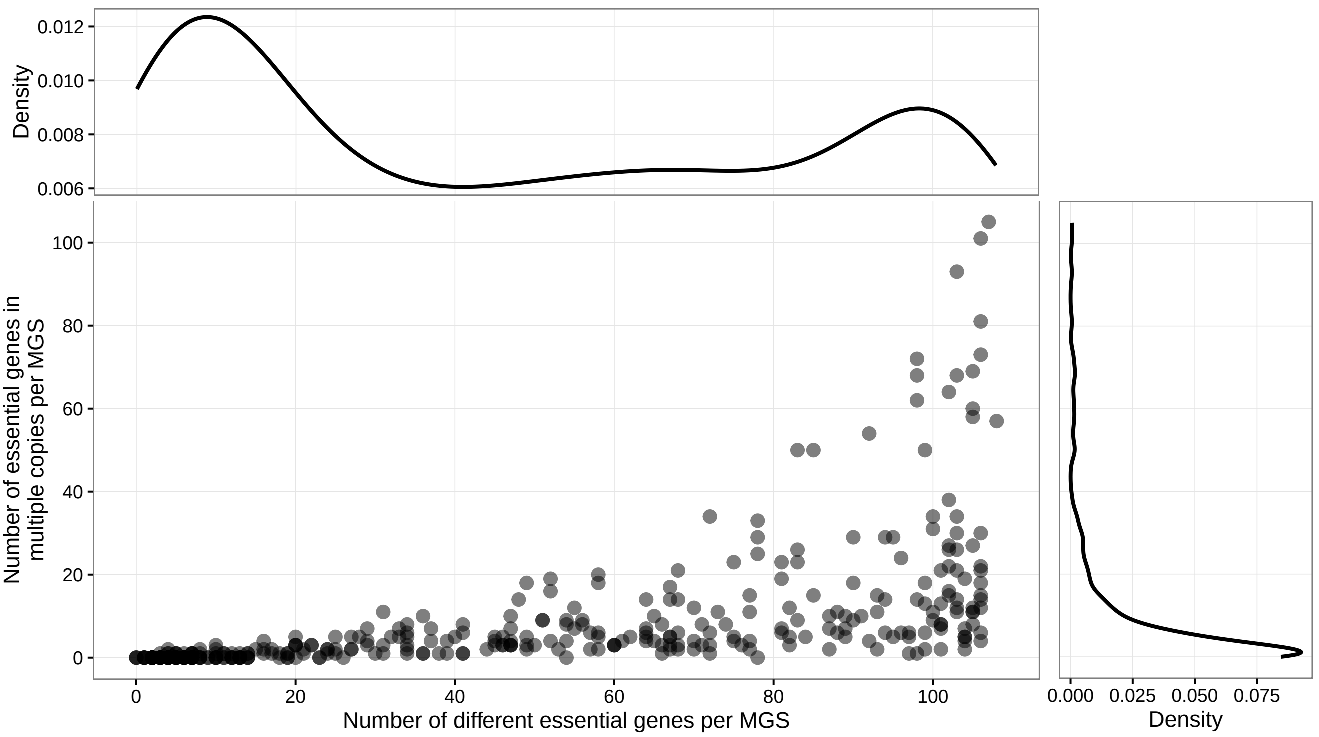

Supplementary Figure 2. Overview of degrees of completeness and contamination for the 365 identified MGS. The degrees of completeness (total numbers of different single- or multi-copy essential genes) are represented on the x-axis and the degrees of contamination (numbers of essential genes in multiple copies) are represented on the y-axis of the scatter plot, per MGS, respectively. Marginal distributions of completeness and contamination are given at the top and at the right of the scatter plot, respectively.

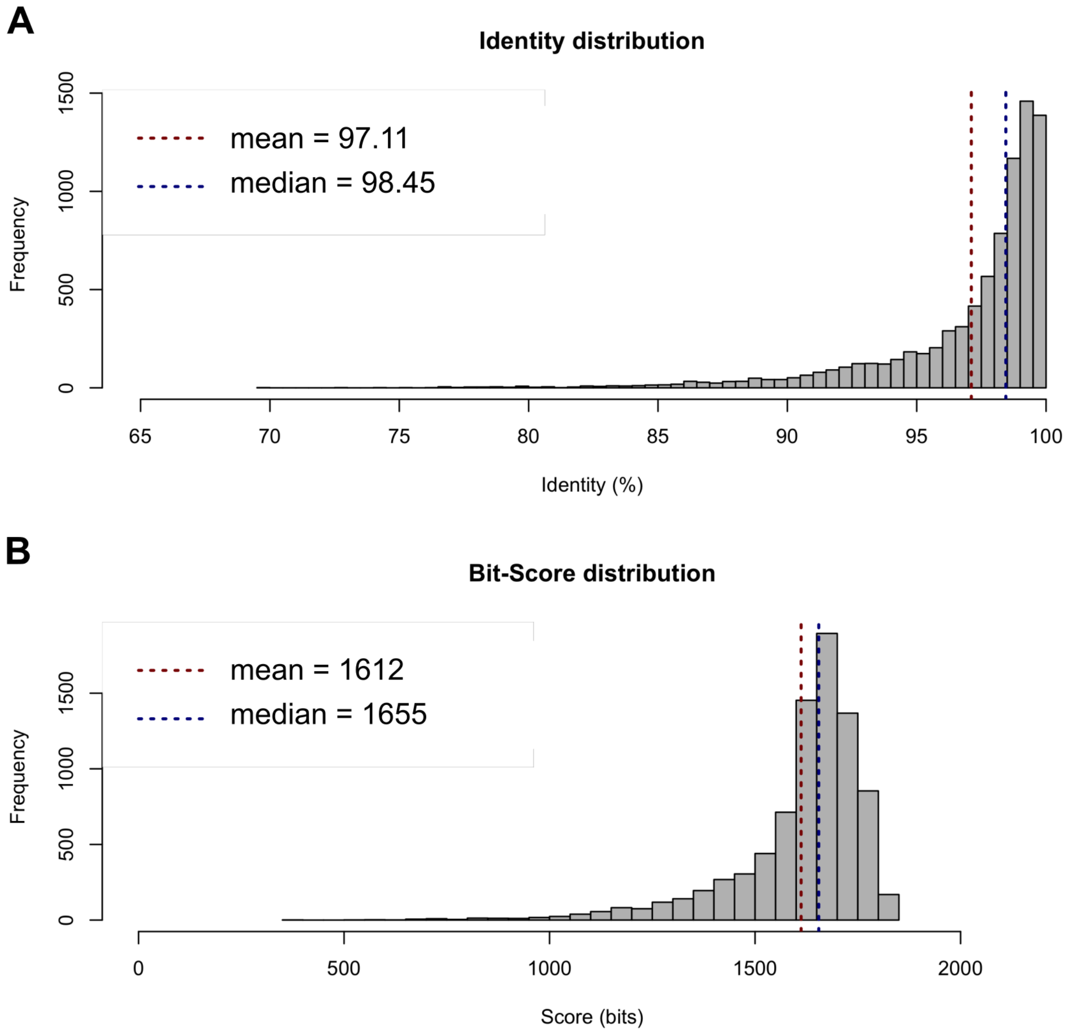

Supplementary Figure 3. Average Nucleotide Identities of the MGS00113-CG01 genome and the MEL.B1 genome. **(A)** Percent identity distribution of the genomic sequences. **(B)** Distribution of the alignment bit scores. Typically, Average Nucleotide Identity values of genomes of the same species are above 95%.

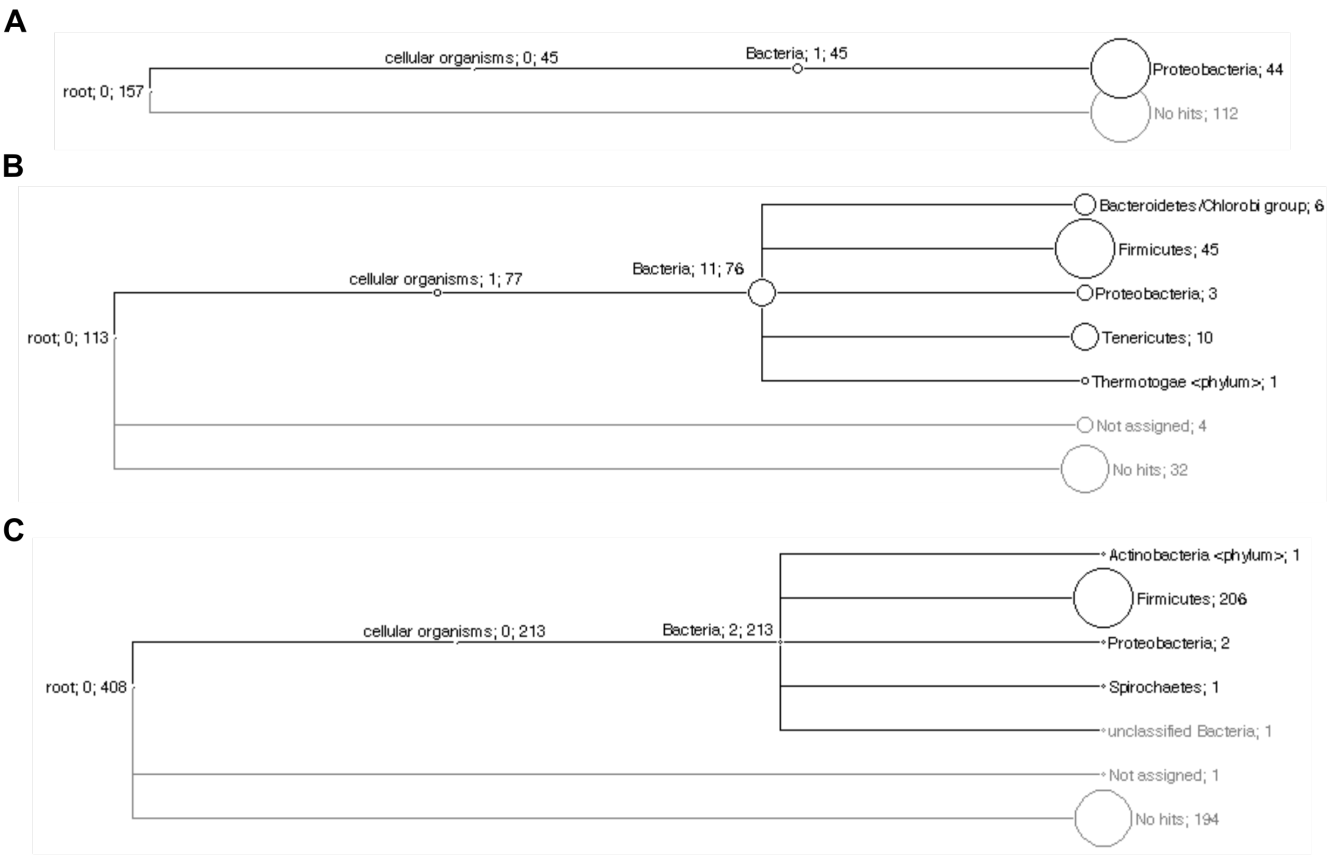

Supplementary Figure 4. Taxonomic assignment of MGS contigs. **(A)** MGS00153. **(B)** MGS00248. **(C)** MGS00113-CG02. **(A) – (C)** The numbers next to the scientific names represent the "Number of sequences summarized" (only for non-leaf nodes) and the "Number of sequences assigned" by BLAST + MEGAN.

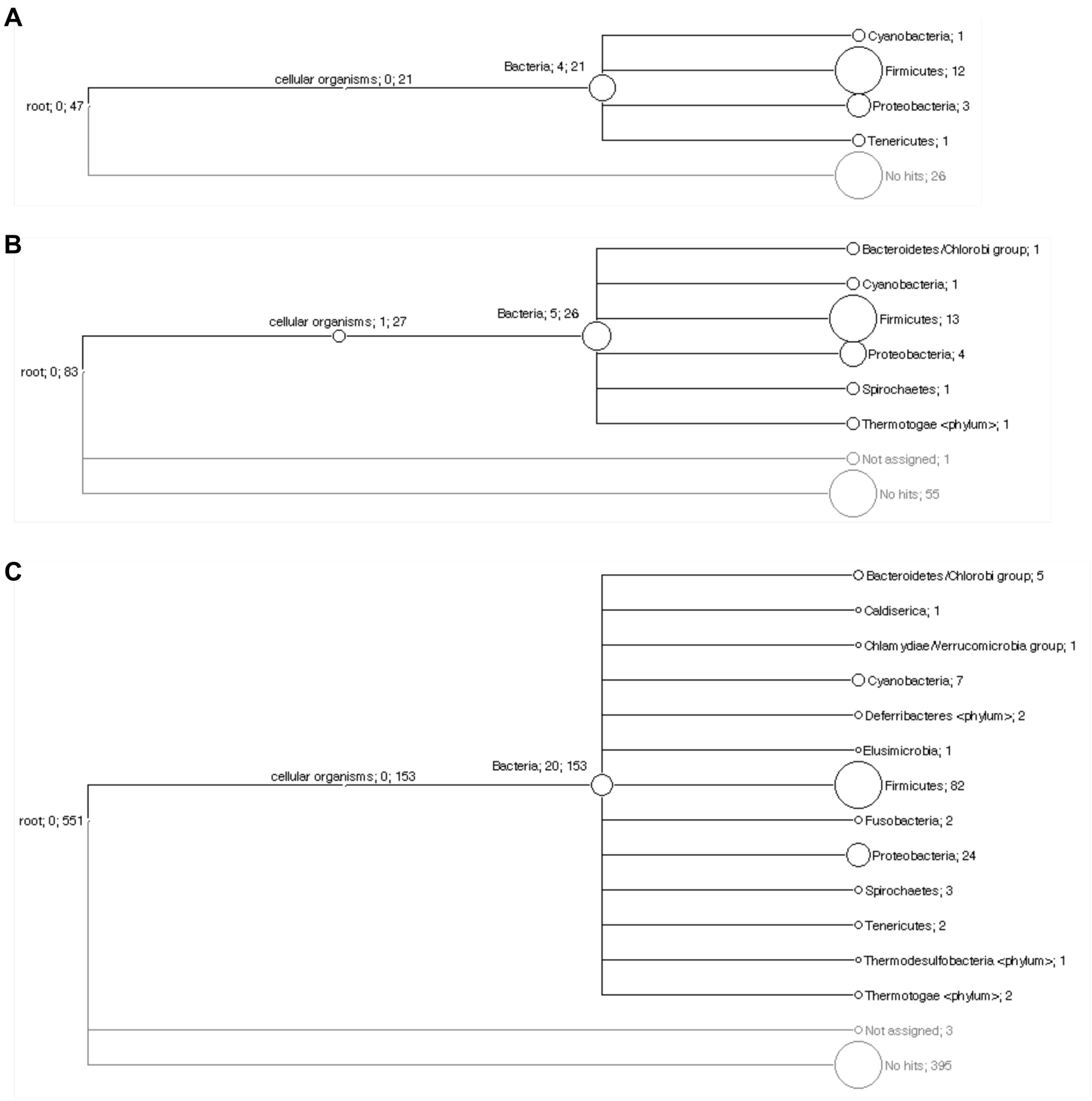

Supplementary Figure 5. Taxonomic assignment of CLSG contigs. **(A)** CLSG01. **(B)** CLSG02. **(C)** CLSG03. **(A)** – **(C)** The numbers next to the scientific names represent the "Number of sequences summarized" (only for non-leaf nodes) and the "Number of sequences assigned" by BLAST + MEGAN.

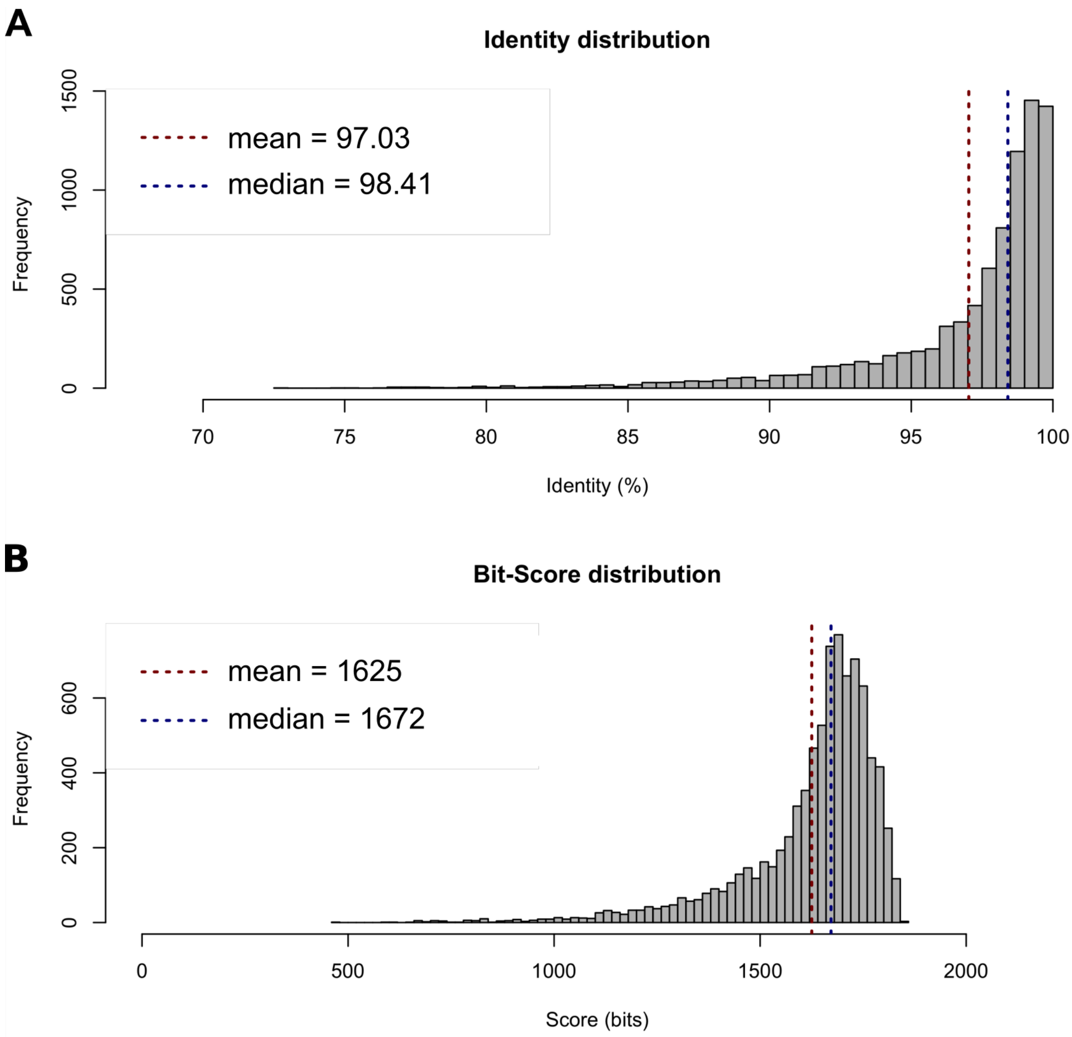

Supplementary Figure 6. Average Nucleotide Identities of the CLSG02 genome and the MEL.B1 genome. **(A)** Percent identity distribution of the genomic sequences. **(B)** Distribution of the alignment bit scores. Typically, Average Nucleotide Identity values of genomes of the same species are above 95%.

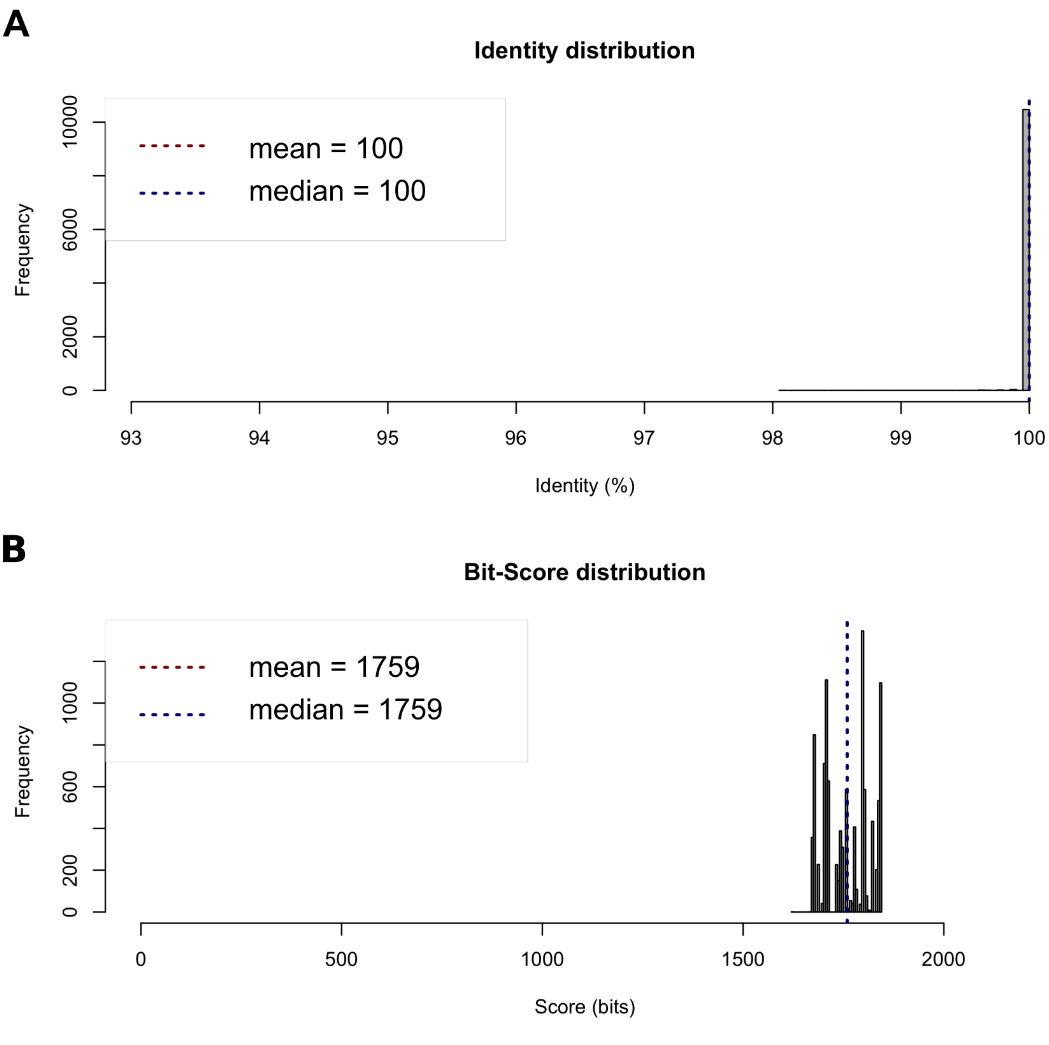

Supplementary Figure 7. Average Nucleotide Identities of the MGS00113-CG01 genome and the CLSG02 genome. **(A)** Percent identity distribution of the genomic sequences. **(B)** Distribution of the alignment bit scores. Typically, Average Nucleotide Identity values of genomes of the same species are above 95%.

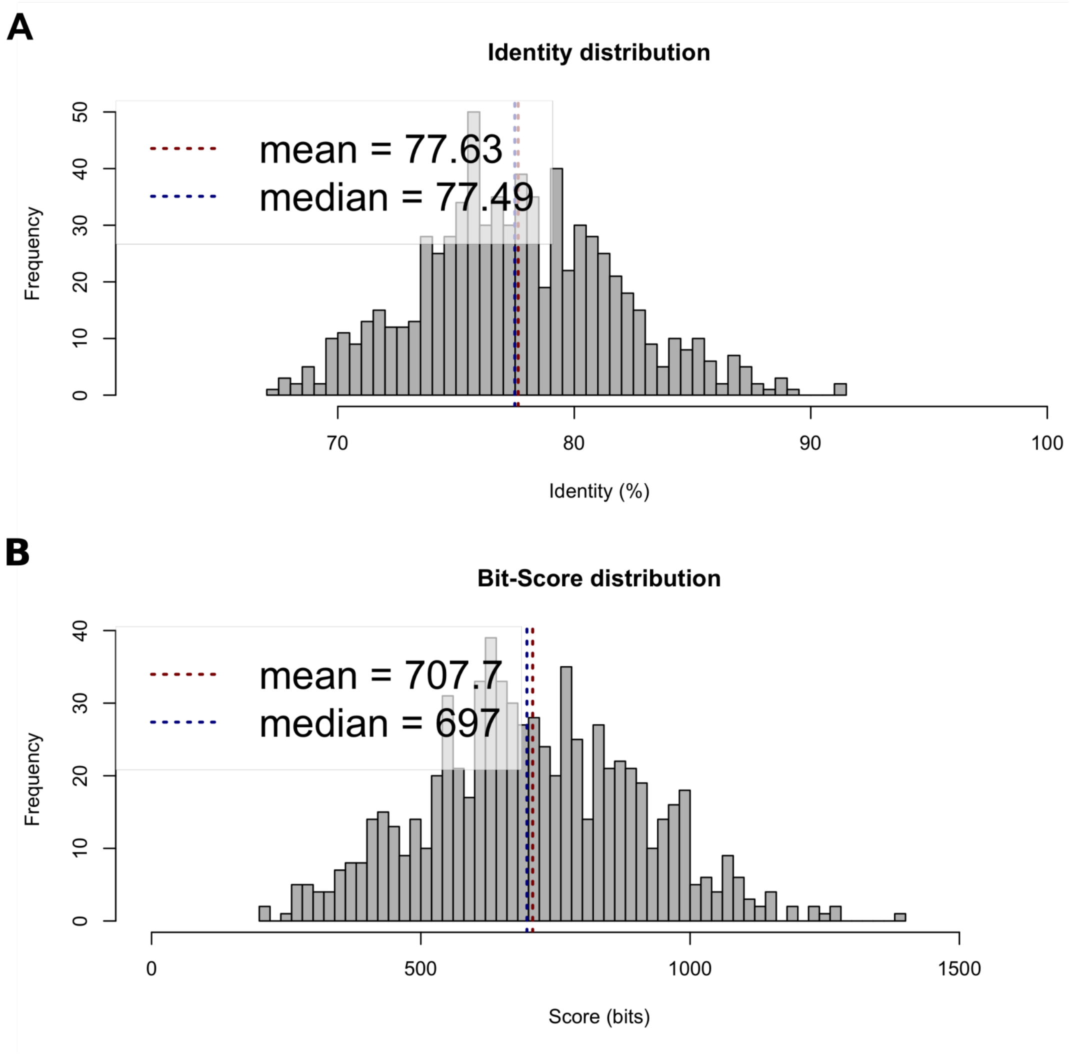

Supplementary Figure 8. Average Nucleotide Identities of the CLSG01 genome and the Zag_111 genome. **(A)** Percent identity distribution of the genomic sequences. **(B)** Distribution of the alignment bit scores. Typically, Average Nucleotide Identity values of genomes of the same species are above 95%.

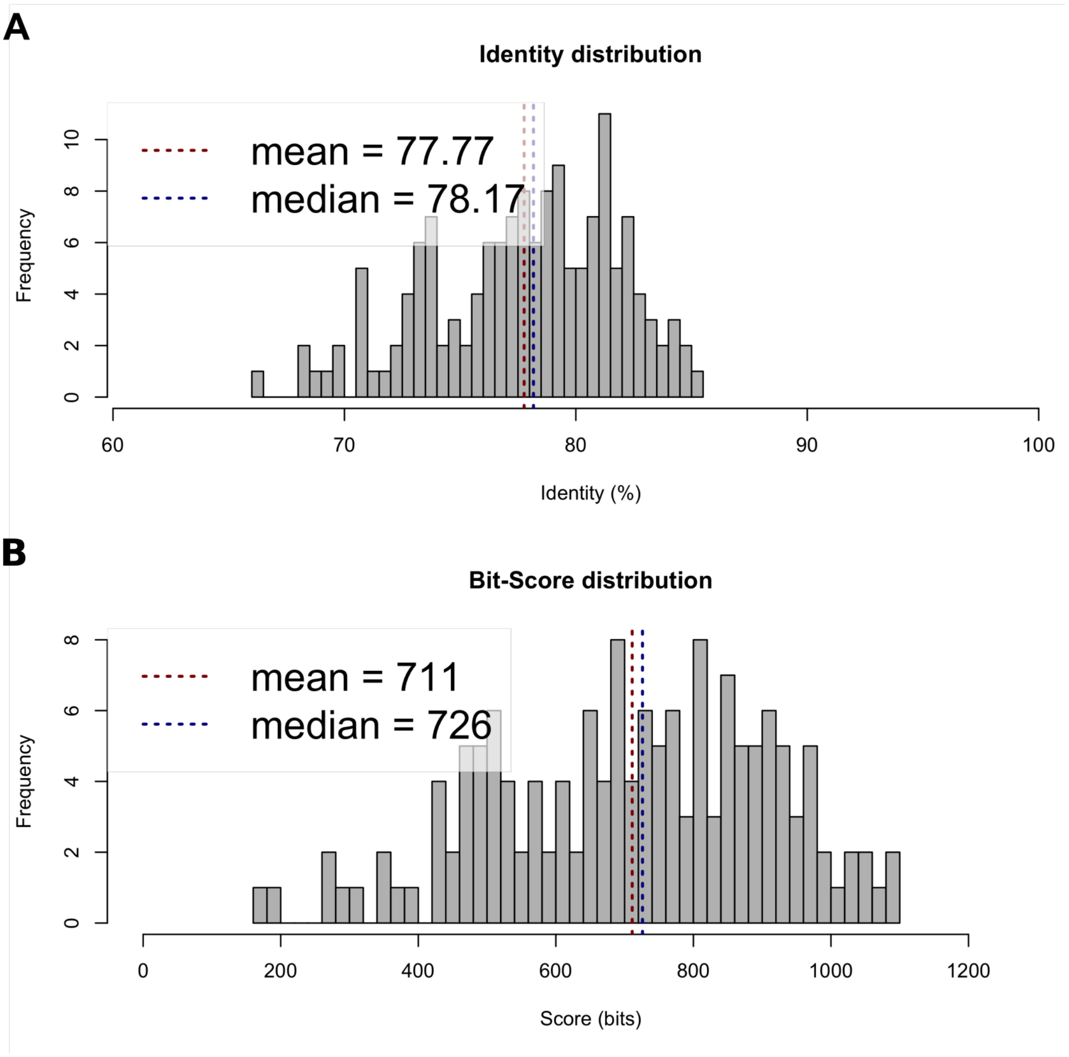

Supplementary Figure 9. Average Nucleotide Identities of the CLSG03 genome and the Zag_221 genome. **(A)** Percent identity distribution of the genomic sequences. **(B)** Distribution of the alignment bit scores. Typically, Average Nucleotide Identity values of genomes of the same species are above 95%.

3 SUPPLEMENTARY TABLES

Supplementary Table 1. Library size and assembly summary statistics over all 55 MUST samples.

	mean	stdev.
# preprocessed paired-end reads	20,862,561	608,594
# contigs	877,215	273,661
Total length	280,853,880	67,955,719
Largest contig length	401,815	140,186
N50	1,024	430

Supplementary Table 2. AMPHORA2 results for MGS00153 – Alphaproteobacteria-like.

Query	Marker	Superkingdom	Phylum	Class	Order	Family	Genus	Species
PROKKA_00639	dnaG	Bacteria(1.00)	Proteobacteria(1.00)	Alphaproteobacteria(1.00)				
PROKKA_01489	frr	Bacteria(1.00)	Proteobacteria(1.00)	Alphaproteobacteria(1.00)	Candidatus Puniceispirillum(0.79)	Candidatus Puniceispirillum marinum(0.79)		
PROKKA_00034	infC	Bacteria(1.00)	Proteobacteria(1.00)	Alphaproteobacteria(1.00)	Candidatus Pelagibacter(1.00)			
PROKKA_01797	nusA	Bacteria(1.00)	Proteobacteria(1.00)	Alphaproteobacteria(1.00)	Candidatus Puniceispirillum(1.00)	Candidatus Puniceispirillum marinum(1.00)		
PROKKA_01545	pgk	Bacteria(1.00)	Proteobacteria(1.00)	Alphaproteobacteria(1.00)				
PROKKA_00682	pyrG	Bacteria(0.96)	Proteobacteria(0.96)	Alphaproteobacteria(0.96)				
PROKKA_00987	rplA	Bacteria(0.95)	Proteobacteria(0.95)	Alphaproteobacteria(0.95)	Candidatus Puniceispirillum(0.47)	Candidatus Puniceispirillum marinum(0.47)		
PROKKA_01817	rplB	Bacteria(1.00)	Proteobacteria(1.00)	Alphaproteobacteria(1.00)	Rhodospirillales(1.00)	Rhodospirillaceae(1.00)	Rhodospirillum(1.00)	Rhodospirillum rubrum(1.00)
PROKKA_01814	rplC	Bacteria(1.00)	Proteobacteria(1.00)	Alphaproteobacteria(1.00)	Rhodospirillales(0.54)	Acetobacteraceae(0.54)		
PROKKA_01815	rplD	Bacteria(0.99)	Proteobacteria(0.99)	Alphaproteobacteria(0.99)	Rhodospirillales(0.99)	Rhodospirillaceae(0.99)	Rhodospirillum(0.99)	Rhodospirillum rubrum(0.99)
PROKKA_01826	rplE	Bacteria(1.00)	Proteobacteria(1.00)	Alphaproteobacteria(1.00)	Sphingomonadales(1.00)	Sphingomonadaceae(1.00)		
PROKKA_01829	rplF	Bacteria(0.96)	Proteobacteria(0.96)	Alphaproteobacteria(0.96)	Rhodospirillales(0.77)			
PROKKA_00988	rplK	Bacteria(0.98)	Proteobacteria(0.98)	Alphaproteobacteria(0.98)	Rhodospirillales(0.35)			
PROKKA_00985	rplL	Bacteria(1.00)	Proteobacteria(1.00)	Alphaproteobacteria(1.00)	Polymorphum gilvum(1.00)			
PROKKA_00582	rplM	Bacteria(0.99)	Proteobacteria(0.99)	Alphaproteobacteria(0.99)	Candidatus Pelagibacter(0.05)			
PROKKA_01824	rplN	Bacteria(0.96)	Proteobacteria(0.96)	Alphaproteobacteria(0.96)	Rhodospirillales(0.64)	Rhodospirillaceae(0.64)	Magnetospirillum(0.42)	Magnetospirillum magneticum(0.42)
PROKKA_01821	rplP	Bacteria(0.96)	Proteobacteria(0.96)	Alphaproteobacteria(0.96)	Rhodospirillales(0.84)	Acetobacteraceae(0.55)		
PROKKA_01443	rplS	Bacteria(0.96)	Proteobacteria(0.96)	Alphaproteobacteria(0.94)	Rhodospirillales(0.43)	Rhodospirillaceae(0.43)	Rhodospirillum(0.43)	Rhodospirillum rubrum(0.43)
PROKKA_00105	rplT	Bacteria(0.96)	Proteobacteria(0.96)	Alphaproteobacteria(0.96)	Rhodospirillales(0.96)	Rhodospirillaceae(0.96)	Rhodospirillum(0.96)	Rhodospirillum rubrum(0.96)
PROKKA_00524	rpmA	Bacteria(0.95)	Proteobacteria(0.95)	Alphaproteobacteria(0.95)	Rhizobiales(0.76)	Rhizobiaceae(0.76)	Candidatus Liberibacter(0.76)	Candidatus Liberibacter asiaticus(0.22)
PROKKA_00983	rpoB	Bacteria(1.00)	Proteobacteria(1.00)	Alphaproteobacteria(1.00)				
PROKKA_01776	rpsB	Bacteria(0.98)	Proteobacteria(0.98)	Alphaproteobacteria(0.98)				
PROKKA_01820	rpsC	Bacteria(0.98)	Proteobacteria(0.98)	Alphaproteobacteria(0.98)	Rhodospirillales(0.48)	Rhodospirillaceae(0.48)	Rhodospirillum(0.48)	Rhodospirillum rubrum(0.48)
PROKKA_01831	rpsE	Bacteria(0.96)	Proteobacteria(0.96)	Alphaproteobacteria(0.96)	Candidatus Puniceispirillum(0.86)	Candidatus Puniceispirillum marinum(0.86)		
PROKKA_00581	rpsI	Bacteria(0.97)	Proteobacteria(0.97)	Alphaproteobacteria(0.97)	Rhodospirillales(0.89)	Acetobacteraceae(0.44)		
PROKKA_01813	rpsJ	Bacteria(0.95)	Proteobacteria(0.95)	Alphaproteobacteria(0.95)	Rhizobiales(0.62)	Phyllobacteriaceae(0.62)	Mesorhizobium(0.62)	Mesorhizobium opportunistum(0.12)
PROKKA_01841	rpsK	Bacteria(0.98)	Proteobacteria(0.98)	Alphaproteobacteria(0.98)	Rhodospirillales(0.76)	Rhodospirillaceae(0.49)	Magnetospirillum(0.34)	Magnetospirillum magneticum(0.34)
PROKKA_01840	rpsM	Bacteria(0.97)	Proteobacteria(0.97)	Alphaproteobacteria(0.39)				
PROKKA_01818	rpsS	Bacteria(0.98)	Proteobacteria(0.98)	Alphaproteobacteria(0.98)	Rhodospirillales(0.98)	Acetobacteraceae(0.39)		
PROKKA_01524	smpB	Bacteria(0.96)	Proteobacteria(0.96)	Alphaproteobacteria(0.96)	Candidatus Pelagibacter(0.84)			
PROKKA_01775	tsf	Bacteria(1.00)	Proteobacteria(1.00)	Alphaproteobacteria(1.00)				

Supplementary Table 3. AMPHORA2 results for MGS00248 – Mollicutes-like.

Query	Marker	Superkingdom	Phylum	Class	Order	Family	Genus	Species
PROKKA_00374	dnaG	Bacteria(0.97)	Tenericutes(0.93)	Mollicutes(0.93)	Mycoplasmatales(0.30)	Mycoplasmataceae(0.30)		
PROKKA_00808	frr	Bacteria(0.97)	Firmicutes(0.71)	Erysipelotrichi(0.71)	Erysipelotrichales(0.71)	Erysipelotrichaceae(0.71)	Erysipelothrix(0.71)	Erysipelothrix rhusiopathiae(0.71)
PROKKA_00143	infC	Bacteria(0.99)	Tenericutes(0.55)	Mollicutes(0.55)				
PROKKA_00455	nusA	Bacteria(1.00)	Firmicutes(1.00)	Erysipelotrichi(1.00)	Erysipelotrichales(1.00)	Erysipelotrichaceae(1.00)	Erysipelothrix(1.00)	Erysipelothrix rhusiopathiae(1.00)
PROKKA_00290	pgk	Bacteria(1.00)	Firmicutes(1.00)	Erysipelotrichi(1.00)	Erysipelotrichales(1.00)	Erysipelotrichaceae(1.00)	Erysipelothrix(1.00)	Erysipelothrix rhusiopathiae(1.00)
PROKKA_01143	rplA	Bacteria(0.99)	Tenericutes(0.99)	Mollicutes(0.99)	Mycoplasmatales(0.90)	Mycoplasmataceae(0.90)		
PROKKA_01142	rplK	Bacteria(0.97)	Tenericutes(0.97)	Mollicutes(0.97)	Mycoplasmatales(0.94)	Mycoplasmataceae(0.94)	Mycoplasma(0.91)	Mycoplasma genitalium(0.14)
PROKKA_01145	rplL	Bacteria(0.95)	Tenericutes(0.91)	Mollicutes(0.91)	Mycoplasmatales(0.91)	Mycoplasmataceae(0.91)	Mycoplasma(0.88)	Mycoplasma penetrans(0.05)
PROKKA_01344	rplM	Bacteria(1.00)	Tenericutes(1.00)	Mollicutes(1.00)	Mycoplasmatales(1.00)	Mycoplasmataceae(1.00)		
PROKKA_00270	rplS	Bacteria(0.95)	Gemmatimonadetes(0.87)	Gemmatimonadetes(0.87)	Gemmatimonadales(0.87)	Gemmatimonadaceae(0.87)	Gemmatimonas(0.87)	Gemmatimonas aurantiaca(0.87)
PROKKA_00141	rplT	Bacteria(0.99)	Tenericutes(0.69)	Mollicutes(0.69)				
PROKKA_00041	rpmA	Bacteria(0.97)	Firmicutes(0.95)	Erysipelotrichi(0.95)	Erysipelotrichales(0.95)	Erysipelotrichaceae(0.95)	Erysipelothrix(0.95)	Erysipelothrix rhusiopathiae(0.95)
PROKKA_01146	rpoB	Bacteria(1.00)	Firmicutes(1.00)	Erysipelotrichi(1.00)	Erysipelotrichales(1.00)	Erysipelotrichaceae(1.00)	Erysipelothrix(1.00)	Erysipelothrix rhusiopathiae(1.00)
PROKKA_01345	rpsI	Bacteria(0.99)	Tenericutes(0.99)	Mollicutes(0.99)	Mycoplasmatales(0.09)	Mycoplasmataceae(0.09)		
PROKKA_01295	rpsK	Bacteria(1.00)	Firmicutes(1.00)	Erysipelotrichi(1.00)	Erysipelotrichales(1.00)	Erysipelotrichaceae(1.00)	Erysipelothrix(1.00)	Erysipelothrix rhusiopathiae(1.00)
PROKKA_01294	rpsM	Bacteria(0.97)	Tenericutes(0.97)	Mollicutes(0.97)	Mycoplasmatales(0.72)	Mycoplasmataceae(0.72)	Mycoplasma(0.11)	
PROKKA_01305	smpB	Bacteria(0.97)	Firmicutes(0.69)	Erysipelotrichi(0.69)	Erysipelotrichales(0.69)	Erysipelotrichaceae(0.69)	Erysipelothrix(0.69)	Erysipelothrix rhusiopathiae(0.69)
PROKKA_00810	tsf	Bacteria(0.99)	Firmicutes(0.99)	Erysipelotrichi(0.99)	Erysipelotrichales(0.99)	Erysipelotrichaceae(0.99)	Erysipelothrix(0.99)	Erysipelothrix rhusiopathiae(0.99)

Supplementary Table 4. AMPHORA2 results for MGS00113-CG02 – Clostridiales-like.

Query	Marker	Superkingdom	Phylum	Class	Order	Family	Genus	Species
PROKKA_02224	dnaG	Bacteria(1.00)	Firmicutes(1.00)	Clostridia(1.00)	Clostridiales(1.00)			
PROKKA_01170	frr	Bacteria(1.00)	Firmicutes(1.00)	Clostridia(1.00)	Clostridiales(1.00)	Eubacteriaceae(1.00)	Eubacterium(1.00)	Eubacterium eligens(1.00)
PROKKA_01518	infC	Bacteria(0.98)	Firmicutes(0.98)	Clostridia(0.98)	Clostridiales(0.98)			
PROKKA_01617	nusA	Bacteria(0.98)	Firmicutes(0.98)	Clostridia(0.98)	Clostridiales(0.98)			
PROKKA_00424	pgk	Bacteria(1.00)	Firmicutes(1.00)	Clostridia(1.00)	Clostridiales(1.00)	Lachnospiraceae(1.00)	Roseburia(1.00)	Roseburia hominis(1.00)
PROKKA_00249	pgk	Bacteria(1.00)	Firmicutes(1.00)	Clostridia(1.00)	Clostridiales(1.00)	Clostridiaceae(1.00)	Clostridium(1.00)	Clostridium sp. SY8519(1.00)
PROKKA_01261	pyrG	Bacteria(1.00)	Firmicutes(1.00)	Clostridia(1.00)	Clostridiales(1.00)	Clostridiaceae(0.88)	Clostridium(0.88)	Clostridium saccharolyticum(0.88)
PROKKA_01273	rplA	Bacteria(1.00)	Firmicutes(1.00)	Clostridia(1.00)	Clostridiales(1.00)	Clostridiaceae(0.69)	Clostridium(0.69)	Clostridium phytofermentans(0.69)
PROKKA_00841	rplB	Bacteria(1.00)	Firmicutes(1.00)	Clostridia(1.00)	Clostridiales(1.00)			
PROKKA_00838	rplC	Bacteria(0.96)	Firmicutes(0.96)	Clostridia(0.96)	Clostridiales(0.96)	Clostridiaceae(0.08)	Clostridium(0.08)	Clostridium phytofermentans(0.08)
PROKKA_00839	rplD	Bacteria(0.97)	Firmicutes(0.97)	Clostridia(0.97)	Clostridiales(0.97)	Eubacteriaceae(0.48)	Eubacterium(0.48)	Eubacterium eligens(0.48)
PROKKA_00850	rplE	Bacteria(0.96)	Firmicutes(0.96)	Clostridia(0.96)	Clostridiales(0.96)	Eubacteriaceae(0.14)	Eubacterium(0.14)	Eubacterium eligens(0.14)
PROKKA_00853	rplF	Bacteria(0.99)	Firmicutes(0.99)	Clostridia(0.99)	Clostridiales(0.99)	Lachnospiraceae(0.82)	Butyrivibrio(0.82)	Butyrivibrio proteoclasticus(0.82)
PROKKA_01274	rplK	Bacteria(0.99)	Firmicutes(0.99)	Clostridia(0.99)	Clostridiales(0.99)	Clostridiaceae(0.33)	Clostridium(0.33)	Clostridium saccharolyticum(0.25)
PROKKA_00509	rplL	Bacteria(0.99)	Firmicutes(0.99)	Clostridia(0.99)	Clostridiales(0.99)	Eubacteriaceae(0.99)	Eubacterium(0.99)	Eubacterium eligens(0.99)
PROKKA_00873	rplM	Bacteria(1.00)	Firmicutes(1.00)	Clostridia(1.00)	Clostridiales(1.00)	Clostridiaceae(1.00)	Clostridium(1.00)	Clostridium phytofermentans(1.00)
PROKKA_00848	rplN	Bacteria(0.99)	Firmicutes(0.99)	Clostridia(0.99)	Clostridiales(0.99)			
PROKKA_00845	rplP	Bacteria(0.97)	Firmicutes(0.97)	Clostridia(0.97)	Clostridiales(0.97)	Clostridiaceae(0.03)	Clostridium(0.03)	Clostridium saccharolyticum(0.03)
PROKKA_02300	rplS	Bacteria(0.99)	Firmicutes(0.99)	Clostridia(0.99)	Clostridiales(0.99)	Lachnospiraceae(0.82)	Butyrivibrio(0.82)	Butyrivibrio proteoclasticus(0.82)
PROKKA_02176	rplS	Bacteria(1.00)	Firmicutes(1.00)	Clostridia(1.00)	Clostridiales(1.00)	Eubacteriaceae(1.00)	Eubacterium(1.00)	Eubacterium rectale(1.00)
PROKKA_01516	rplT	Bacteria(0.97)	Firmicutes(0.97)	Clostridia(0.97)	Clostridiales(0.97)	Eubacteriaceae(0.57)	Eubacterium(0.57)	Eubacterium rectale(0.57)
PROKKA_01035	rpmA	Bacteria(1.00)	Firmicutes(1.00)	Clostridia(1.00)	Clostridiales(1.00)	Lachnospiraceae(0.29)	Butyrivibrio(0.14)	Butyrivibrio proteoclasticus(0.14)
PROKKA_00510	rpoB	Bacteria(0.95)	Firmicutes(0.95)	Clostridia(0.95)	Clostridiales(0.95)			
PROKKA_01609	rpsB	Bacteria(1.00)	Firmicutes(1.00)	Clostridia(1.00)	Clostridiales(1.00)			
PROKKA_00844	rpsC	Bacteria(1.00)	Firmicutes(1.00)	Clostridia(1.00)	Clostridiales(1.00)	Lachnospiraceae(1.00)	Butyrivibrio(1.00)	Butyrivibrio proteoclasticus(1.00)
PROKKA_00855	rpsE	Bacteria(0.99)	Firmicutes(0.99)	Clostridia(0.99)	Clostridiales(0.99)	Clostridiaceae(0.94)	Clostridium(0.94)	Clostridium phytofermentans(0.94)
PROKKA_00874	rpsI	Bacteria(0.96)	Firmicutes(0.96)	Clostridia(0.96)	Clostridiales(0.96)	Clostridiaceae(0.80)	Clostridium(0.80)	Clostridium phytofermentans(0.60)
PROKKA_00837	rpsJ	Bacteria(1.00)	Firmicutes(1.00)	Clostridia(1.00)	Clostridiales(1.00)	Eubacteriaceae(1.00)	Eubacterium(1.00)	Eubacterium eligens(1.00)
PROKKA_00863	rpsK	Bacteria(0.99)	Firmicutes(0.99)	Clostridia(0.99)	Clostridiales(0.99)	Eubacteriaceae(0.30)	Eubacterium(0.30)	Eubacterium eligens(0.30)
PROKKA_00862	rpsM	Bacteria(0.99)	Firmicutes(0.99)	Clostridia(0.99)	Clostridiales(0.99)	Eubacteriaceae(0.92)	Eubacterium(0.92)	Eubacterium eligens(0.92)
PROKKA_00842	rpsS	Bacteria(0.98)	Firmicutes(0.98)	Clostridia(0.98)	Clostridiales(0.98)	Lachnospiraceae(0.56)	Butyrivibrio(0.48)	Butyrivibrio proteoclasticus(0.48)
PROKKA_01401	smpB	Bacteria(0.96)	Firmicutes(0.96)	Clostridia(0.96)	Clostridiales(0.96)	Clostridiaceae(0.18)	Clostridium(0.18)	Clostridium phytofermentans(0.18)
PROKKA_00435	smpB	Bacteria(0.99)	Firmicutes(0.99)	Clostridia(0.99)	Clostridiales(0.99)	Eubacteriaceae(0.99)	Eubacterium(0.99)	Eubacterium rectale(0.99)
PROKKA_01610	tsf	Bacteria(0.97)	Firmicutes(0.97)	Clostridia(0.97)	Clostridiales(0.97)			

Supplementary Table 5. AMPHORA2 results for CLSG01.

Query	Marker	Superkingdom	Phylum	Class	Order	Family	Genus	Species
PROKKA_01710	dnaG	Bacteria(1.00)	Firmicutes(1.00)	Clostridia(1.00)	Natranaerobiales(0.80)	Natranaerobiaceae(0.80)	Natranaerobius(0.80)	Natranaerobius thermophilus(0.80)
PROKKA_01499	frr	Bacteria(0.96)	Firmicutes(0.70)	Clostridia(0.64)	Clostridiales(0.64)	Ruminococcaceae(0.39)	Ethanoligenens(0.13)	Ethanoligenens harbinense(0.13)
PROKKA_01276	infC	Bacteria(0.97)	Cyanobacteria(0.95)					
PROKKA_00524	nusA	Bacteria(0.96)	Cyanobacteria(0.17)					
PROKKA_00736	pgk	Bacteria(1.00)	Bacteroidetes(0.90)					
PROKKA_01566	pyrG	Bacteria(0.98)	Cyanobacteria(0.90)					
PROKKA_01591	rplA	Bacteria(0.99)	Firmicutes(0.99)	Clostridia(0.99)	Thermoanaerobacterales(0.99)	Thermodesulfobiaceae(0.99)	Thermodesulfobium(0.99)	Thermodesulfobium narugense(0.99)
PROKKA_00874	rplB	Bacteria(1.00)	Cyanobacteria(1.00)					
PROKKA_00871	rplC	Bacteria(1.00)	Cyanobacteria(1.00)					
PROKKA_00872	rplD	Bacteria(1.00)	Cyanobacteria(1.00)					
PROKKA_00883	rplE	Bacteria(0.96)	Firmicutes(0.96)	Clostridia(0.95)	Clostridiales(0.81)	Symbiobacterium(0.43)	Symbiobacterium thermophilum(0.43)	
PROKKA_00886	rplF	Bacteria(1.00)	Spirochaetes(1.00)	Spirochaetia(1.00)	Spirochaetales(1.00)	Leptospiraceae(1.00)	Leptospira(1.00)	
PROKKA_01592	rplK	Bacteria(1.00)	Spirochaetes(1.00)	Spirochaetia(1.00)	Spirochaetales(1.00)	Leptospiraceae(1.00)	Leptospira(1.00)	
PROKKA_00324	rplL	Bacteria(1.00)	Cyanobacteria(1.00)					
PROKKA_00019	rplM	Bacteria(0.97)	Actinobacteria(0.97)	Actinobacteria(0.97)	Coriobacteriales(0.97)	Coriobacteriaceae(0.97)		
PROKKA_00881	rplN	Bacteria(1.00)	Cyanobacteria(1.00)					
PROKKA_00878	rplP	Bacteria(1.00)	Fibrobacteres(1.00)	Fibrobacteria(1.00)	Fibrobacterales(1.00)	Fibrobacteraceae(1.00)	Fibrobacter(1.00)	Fibrobacter succinogenes(1.00)
PROKKA_01095	rplS	Bacteria(1.00)	Cyanobacteria(1.00)					
PROKKA_01140	rplT	Bacteria(0.96)	Cyanobacteria(0.81)					
PROKKA_00330	rpmA	Bacteria(0.95)	Cyanobacteria(0.87)	Chroococcales(0.11)	Cyanothece(0.08)	Cyanothece sp. PCC 7424(0.01)		
PROKKA_00576	rpoB	Bacteria(1.00)	Cyanobacteria(1.00)					
PROKKA_00301	rpsB	Bacteria(1.00)	Thermotogae(1.00)	Thermotogae(1.00)	Thermotogales(1.00)	Thermotogaceae(1.00)		
PROKKA_00877	rpsC	Bacteria(1.00)	Cyanobacteria(1.00)					
PROKKA_01533	rpsE	Bacteria(0.97)	Cyanobacteria(0.97)					
PROKKA_00018	rpsI	Bacteria(0.95)	Elusimicrobia(0.63)	Elusimicrobia(0.63)	Elusimicrobiales(0.63)	Elusimicrobiaceae(0.63)	Elusimicrobium(0.63)	Elusimicrobium minutum(0.63)
PROKKA_00870	rpsJ	Bacteria(0.99)	Cyanobacteria(0.99)					
PROKKA_00024	rpsK	Bacteria(0.98)	Cyanobacteria(0.86)					
PROKKA_00025	rpsM	Bacteria(0.95)	Actinobacteria(0.83)	Actinobacteria(0.83)	Actinomycetales(0.83)	Nakamurellaceae(0.64)	Nakamurella(0.64)	Nakamurella multipartita(0.64)
PROKKA_00875	rpsS	Bacteria(0.95)	Thermotogae(0.50)	Thermotogae(0.50)	Thermotogales(0.50)	Thermotogaceae(0.50)		
PROKKA_01463	smpB	Bacteria(0.97)	Firmicutes(0.97)	Bacilli(0.97)	Bacillales(0.97)	Alicyclobacillaceae(0.97)	Kyrpidia(0.88)	Kyrpidia tusciae(0.88)
PROKKA_00300	tsf	Bacteria(1.00)						

Supplementary Table 6. AMPHORA2 results for CLSG02.

Query	Marker	Superkingdom	Phylum	Class	Order	Family	Genus	Species
PROKKA_00807	dnaG	Bacteria(0.98)	Cyanobacteria(0.98)					
PROKKA_00168	frr	Bacteria(0.95)	Firmicutes(0.58)	Clostridia(0.36)	Clostridiales(0.36)	Ruminococcaceae(0.34)	Ethanoligenens(0.03)	Ethanoligenens harbinense(0.03)
PROKKA_00150	infC	Bacteria(0.98)	Spirochaetes(0.98)	Spirochaetia(0.98)	Spirochaetales(0.98)	Leptospiraceae(0.98)	Leptospira(0.98)	
PROKKA_02114	nusA	Bacteria(0.99)	Fusobacteria(0.89)	Fusobacteriia(0.89)	Fusobacteriales(0.89)			
PROKKA_01439	pgk	Bacteria(0.97)	Bacteroidetes(0.65)					
PROKKA_00510	pyrG	Bacteria(0.99)	Fusobacteria(0.99)	Fusobacteriia(0.99)	Fusobacteriales(0.99)			
PROKKA_02032	rplA	Bacteria(1.00)	Firmicutes(1.00)	Clostridia(1.00)	Thermoanaerobacterales(1.00)	Thermodesulfobiaceae(1.00)	Thermodesulfobium(1.00)	Thermodesulfobium narugense(1.00)
PROKKA_00440	rplB	Bacteria(0.99)	Cyanobacteria(0.99)					
PROKKA_00443	rplC	Bacteria(1.00)	Cyanobacteria(1.00)					
PROKKA_00442	rplD	Bacteria(1.00)	Cyanobacteria(1.00)					
PROKKA_00431	rplE	Bacteria(0.96)	Firmicutes(0.81)	Clostridia(0.73)	Clostridiales(0.73)	Symbiobacterium(0.68)	Symbiobacterium thermophilum(0.68)	
PROKKA_00424	rplF	Bacteria(0.95)	Proteobacteria(0.95)	Epsilonproteobacteria(0.95)				
PROKKA_02033	rplK	Bacteria(1.00)	Spirochaetes(1.00)	Spirochaetia(1.00)	Spirochaetales(1.00)	Leptospiraceae(1.00)	Leptospira(1.00)	
PROKKA_01684	rplL	Bacteria(0.99)	Cyanobacteria(0.99)					
PROKKA_01606	rplM	Bacteria(0.99)	Tenericutes(0.99)	Mollicutes(0.99)	Mycoplasmatales(0.67)	Mycoplasmataceae(0.67)	Mycoplasma(0.14)	
PROKKA_00433	rplN	Bacteria(1.00)	Cyanobacteria(1.00)					
PROKKA_00436	rplP	Bacteria(0.99)	Fibrobacteres(0.99)	Fibrobacteria(0.99)	Fibrobacterales(0.99)	Fibrobacteraceae(0.99)	Fibrobacter(0.99)	Fibrobacter succinogenes(0.99)
PROKKA_00667	rplS	Bacteria(1.00)	Cyanobacteria(1.00)					
PROKKA_01459	rplT	Bacteria(1.00)	Cyanobacteria(1.00)					
PROKKA_00086	rpmA	Bacteria(0.96)	Cyanobacteria(0.95)	Chroococcales(0.62)	Cyanothece(0.37)			
PROKKA_02130	rpoB	Bacteria(1.00)	Cyanobacteria(1.00)					
PROKKA_01490	rpsB	Bacteria(1.00)	Thermotogae(1.00)	Thermotogae(1.00)	Thermotogales(1.00)	Thermotogaceae(1.00)		
PROKKA_00437	rpsC	Bacteria(1.00)	Cyanobacteria(1.00)					
PROKKA_00464	rpsE	Bacteria(0.95)	Cyanobacteria(0.95)					
PROKKA_01607	rpsI	Bacteria(0.95)	Spirochaetes(0.48)	Spirochaetia(0.48)	Spirochaetales(0.48)	Brachyspiraceae(0.40)	Brachyspira(0.40)	
PROKKA_00444	rpsJ	Bacteria(0.99)	Firmicutes(0.93)	Clostridia(0.93)	Thermoanaerobacterales(0.93)	Thermodesulfobiaceae(0.93)	Coprothermobacter(0.93)	Coprothermobacter proteolyticus(0.93)
PROKKA_01601	rpsK	Bacteria(0.99)	Cyanobacteria(0.92)					
PROKKA_01600	rpsM	Bacteria(0.95)	Actinobacteria(0.95)	Actinobacteria(0.95)	Actinomycetales(0.95)	Corynebacteriaceae(0.95)	Corynebacterium(0.95)	Corynebacterium kroppenstedtii(0.86)
PROKKA_00439	rpsS	Bacteria(0.97)	Chlorobi(0.97)	Chlorobia(0.97)	Chlorobiales(0.97)	Chlorobiaceae(0.97)		
PROKKA_00177	smpB	Bacteria(0.95)	Firmicutes(0.87)	Bacilli(0.55)	Bacillales(0.55)	Alicyclobacillaceae(0.55)	Kyrpidia(0.55)	Kyrpidia tusciae(0.55)
PROKKA_01491	tsf	Bacteria(1.00)						

Supplementary Table 7. AMPHORA2 results for CLSG03.

Query	Marker	Superkingdom	Phylum	Class	Order	Family	Genus	Species
PROKKA_00832	dnaG	Bacteria(1.00)	Cyanobacteria(0.80)					
PROKKA_00921	frr	Bacteria(0.96)	Firmicutes(0.96)	Clostridia(0.96)	Clostridiales(0.96)	Ruminococcaceae(0.15)		
PROKKA_00282	pgk	Bacteria(1.00)	Bacteroidetes(1.00)					
PROKKA_00377	pyrG	Bacteria(0.96)	Fibrobacteres(0.78)	Fibrobacteria(0.78)	Fibrobacterales(0.78)	Fibrobacteraceae(0.78)	Fibrobacter(0.78)	Fibrobacter succinogenes(0.78)
PROKKA_01681	rplA	Bacteria(1.00)	Firmicutes(1.00)	Clostridia(1.00)	Clostridiales(1.00)	Ruminococcaceae(1.00)		
PROKKA_00527	rplA	Bacteria(1.00)	Firmicutes(1.00)	Clostridia(1.00)	Thermoanaerobacterales(1.00)	Thermodesulfobiaceae(1.00)	Thermodesulfobium(1.00)	Thermodesulfobium narugense(1.00)
PROKKA_01019	rplC	Bacteria(1.00)	Cyanobacteria(1.00)					
PROKKA_01018	rplD	Bacteria(1.00)	Cyanobacteria(1.00)					
PROKKA_00683	rplE	Bacteria(0.95)	Proteobacteria(0.25)	Deltaproteobacteria(0.25)	Desulfobacterales(0.24)	Desulfobacteraceae(0.24)	Desulfatibacillum(0.22)	Desulfatibacillum alkenivorans(0.22)
PROKKA_00686	rplF	Bacteria(0.98)	Deferribacteres(0.24)	Deferribacteres(0.24)	Deferribacterales(0.24)	Deferribacteraceae(0.24)		
PROKKA_00526	rplK	Bacteria(1.00)	Spirochaetes(1.00)	Spirochaetia(1.00)	Spirochaetales(1.00)	Leptospiraceae(1.00)	Leptospira(1.00)	
PROKKA_01328	rplL	Bacteria(0.99)	Spirochaetes(0.99)	Spirochaetia(0.99)	Spirochaetales(0.99)	Leptospiraceae(0.99)	Leptospira(0.99)	
PROKKA_00177	rplM	Bacteria(0.95)	Actinobacteria(0.67)	Actinobacteria(0.67)	Actinomycetales(0.67)	Micrococcaceae(0.21)	Micrococcus(0.02)	Micrococcus luteus(0.02)
PROKKA_00681	rplN	Bacteria(1.00)	Cyanobacteria(1.00)					
PROKKA_00678	rplP	Bacteria(0.99)	Fibrobacteres(0.99)	Fibrobacteria(0.99)	Fibrobacterales(0.99)	Fibrobacteraceae(0.99)	Fibrobacter(0.99)	Fibrobacter succinogenes(0.99)
PROKKA_00294	rplS	Bacteria(1.00)	Cyanobacteria(1.00)					
PROKKA_01228	rpmA	Bacteria(0.95)	Cyanobacteria(0.84)	Chroococcales(0.12)	Cyanothece(0.10)	Cyanothece sp. PCC 7822(0.01)		
PROKKA_00194	rpoB	Bacteria(1.00)	Cyanobacteria(1.00)					
PROKKA_00466	rpsB	Bacteria(0.96)	Thermotogae(0.96)	Thermotogae(0.96)	Thermotogales(0.96)	Thermotogaceae(0.96)		
PROKKA_00677	rpsC	Bacteria(1.00)	Cyanobacteria(1.00)					
PROKKA_01051	rpsE	Bacteria(1.00)	Cyanobacteria(1.00)					
PROKKA_00178	rpsI	Bacteria(0.95)	Spirochaetes(0.85)	Spirochaetia(0.85)	Spirochaetales(0.85)	Leptospiraceae(0.54)	Leptospira(0.54)	
PROKKA_01020	rpsJ	Bacteria(0.96)	Cyanobacteria(0.96)	Chroococcales(0.15)	Acaryochloris(0.05)	Acaryochloris marina(0.05)		
PROKKA_01152	rpsK	Bacteria(0.98)	Cyanobacteria(0.87)					
PROKKA_01151	rpsM	Bacteria(0.95)	Actinobacteria(0.48)	Actinobacteria(0.48)	Actinomycetales(0.48)	Corynebacteriaceae(0.28)	Corynebacterium(0.28)	
PROKKA_00675	rpsS	Bacteria(0.95)	Proteobacteria(0.85)	Gammaproteobacteria(0.85)	Alteromonadales(0.64)	Idiomarinaceae(0.50)	Idiomarina(0.50)	Idiomarina loihiensis(0.50)
PROKKA_01295	smpB	Bacteria(0.95)	Firmicutes(0.95)	Clostridia(0.74)	Natranaerobiales(0.07)	Natranaerobiaceae(0.07)	Natranaerobius(0.07)	Natranaerobius thermophilus(0.07)
PROKKA_00467	tsf	Bacteria(1.00)						

REFERENCES

Atarashi, K., Tanoue, T., Oshima, K., Suda, W., Nagano, Y., Nishikawa, H., et al. (2013). Treg induction by a rationally selected mixture of Clostridia strains from the human microbiota. *Nature* 500, 232–236. doi:10.1038/nature12331

Biegel, E., Schmidt, S., González, J. M., and Müller, V. (2011). Biochemistry, evolution and physiological function of the Rnf complex, a novel ion-motive electron transport complex in prokaryotes. *Cellular and molecular life sciences* 68, 613–634

Canani, R. B., Costanzo, M. D., Leone, L., Pedata, M., Meli, R., and Calignano, A. (2011). Potential beneficial effects of butyrate in intestinal and extraintestinal diseases. *World Journal of Gastroenterology* 17, 1519–1528. doi:10.3748/wjg.v17.i12.1519

Chang, P. V., Hao, L., Offermanns, S., and Medzhitov, R. (2014). The microbial metabolite butyrate regulates intestinal macrophage function via histone deacetylase inhibition. *Proceedings of the National Academy of Sciences of the United States of America* 111, 2247–2252. doi:10.1073/pnas.1322269111

Christensen-Dalsgaard, M., Jø rgensen, M. G., and Gerdes, K. (2010). Three new RelE-homologous mRNA interferases of *Escherichia coli* differentially induced by environmental stresses. *Molecular Microbiology* 75, 333–348. doi:10.1111/j.1365-2958.2009.06969.x

Di Rienzi, S. C., Sharon, I., Wrighton, K. C., Koren, O., Hug, L. a., Thomas, B. C., et al. (2013). The human gut and groundwater harbor non-photosynthetic bacteria belonging to a new candidate phylum sibling to Cyanobacteria. *eLife* 2013, 1–25. doi:10.7554/eLife.01102.001

Fozo, E. M., Makarova, K. S., Shabalina, S. a., Yutin, N., Koonin, E. V., and Storz, G. (2010). Abundance of type I toxin-antitoxin systems in bacteria: Searches for new candidates and discovery of novel families. *Nucleic Acids Research* 38, 3743–3759. doi:10.1093/nar/gkq054

Furusawa, Y., Obata, Y., Fukuda, S., Endo, T. a., Nakato, G., Takahashi, D., et al. (2013). Commensal microbe-derived butyrate induces the differentiation of colonic regulatory T cells. *Nature* 504, 446–450. doi:10.1038/nature12721

Hayes, F. (2003). Toxins-antitoxins: plasmid maintenance, programmed cell death, and cell cycle arrest. *Science* 301, 1496–1499. doi:10.1126/science.1088157

Johansson, K.-E. and Pettersson, B. (2002). Taxonomy of Mollicutes. In *Molecular Biology and Pathogenicity of Mycoplasmas*, eds. S. Razin and R. Herrmann (Boston, MA: Springer US). 1 – 29. doi:10.1007/b113360

Laczny, C. C., Pinel, N., Vlassis, N., and Wilmes, P. (2014). Alignment-free visualization of metagenomic data by nonlinear dimension reduction. *Scientific Reports* 4, 4516. doi:10.1038/srep04516

Ludwig, W., Schleifer, K.-H., and Whitman, W. (2009). Revised road map to the phylum Firmicutes. *Bergeys Manual of Systematic Bacteriology* 3, 1–13. doi:10.1007/978-0-387-68489-5_1

Meehan, C. J. and Beiko, R. G. (2014). A phylogenomic view of ecological specialization in the lachnospiraceae, a family of digestive tract-associated bacteria. *Genome Biology and Evolution* 6, 703–713. doi:10.1093/gbe/evu050

Miller, C. S., Baker, B. J., Thomas, B. C., Singer, S. W., and Banfield, J. F. (2011). EMIRGE: reconstruction of full-length ribosomal genes from microbial community short read sequencing data. *Genome Biology* 12, R44. doi:10.1186/gb-2011-12-5-r44

Pandey, D. P. and Gerdes, K. (2005). Toxin-antitoxin loci are highly abundant in free-living but lost from host-associated prokaryotes. *Nucleic Acids Research* 33, 966–976. doi:10.1093/nar/gki201

Soo, R. M., Skennerton, C. T., Sekiguchi, Y., Imelfort, M., Paech, S. J., Dennis, P. G., et al. (2014). An expanded genomic representation of the phylum Cyanobacteria. *Genome Biology and Evolution* 6, 1031–1045. doi:10.1093/gbe/evu073

Yadav, H., Lee, J. H., Lloyd, J., Walter, P., and Rane, S. G. (2013). Beneficial metabolic effects of a probiotic via butyrate-induced GLP-1 hormone secretion. *Journal of Biological Chemistry* 288, 25088–25097. doi:10.1074/jbc.M113.452516

Yamaguchi, Y., Park, J.-H., and Inouye, M. (2011). Toxin-antitoxin systems in bacteria and archaea. *Annual Review of Genetics* 45, 61–79. doi:10.1146/annurev-genet-110410-132412

Zimmerman, M. a., Singh, N., Martin, P. M., Thangaraju, M., Ganapathy, V., Waller, J. L., et al. (2012). Butyrate suppresses colonic inflammation through HDAC1-dependent Fas upregulation and Fas-mediated apoptosis of T cells. *AJP: Gastrointestinal and Liver Physiology* 302, G1405–G1415. doi:10.1152/ajpgi.00543.2011
